# Sleep duration mediates the association between heavy metals and the prevalence of depression: an integrated approach from the NHANES (2005–2020)

**DOI:** 10.3389/fpsyt.2024.1455896

**Published:** 2024-09-02

**Authors:** Qingsong Mao, Xiaoyi Zhu, Yuzhe Kong

**Affiliations:** ^1^ Hepatobiliary Pancreatic Surgery, Banan Hospital of Chongqing Medical University, Chongqing, China; ^2^ Xiangya School of Medicine, Central South University, Changsha, China

**Keywords:** sleep duration, heavy metal, depression, mediation analysis, national health and nutrition examination survey

## Abstract

**Purpose:**

This study investigates the association between heavy metal exposure and the prevalence of depression, with the mediating role of sleep duration.

**Method:**

Our study investigated the association between heavy metal concentration and depression risk in the NHANES from 2005 to 2020. We used logistic regression analysis, WQS regression model and BKMR model to assess the association. Mediation analysis was performed to explore the role of sleep duration in heavy metal exposure-induced depression.

**Result:**

Of the 10,825 individuals included in this study, 705 (6.85%) were diagnosed with depression. We found a positive association between the cadmium (Cd), cobalt (Co), lead (Pb), antimony (Sb), wolfram (W) concentration and the prevalence of depression. Moreover, heavy metals mixtures were positively correlated with the prevalence of depression, with Cd identified as the main contributor in the WQS regression model (0.4654). Furthermore, sleep duration mediated the association between Cd, molybdenum (Mo), Pb, Sb, W exposure and prevalence of depression, explaining 3.12%, 15.84%, 18.24%, 9.56%, 3.68% of the effect.

**Conclusion:**

The findings affirm that heavy metal exposure is linked to higher depression incidence and that this relationship is partly mediated by sleep duration alterations. The study underscores the importance of environmental health monitoring and interventions aimed at reducing heavy metal exposure to mitigate its psychological impacts.

## Graphical Abstract

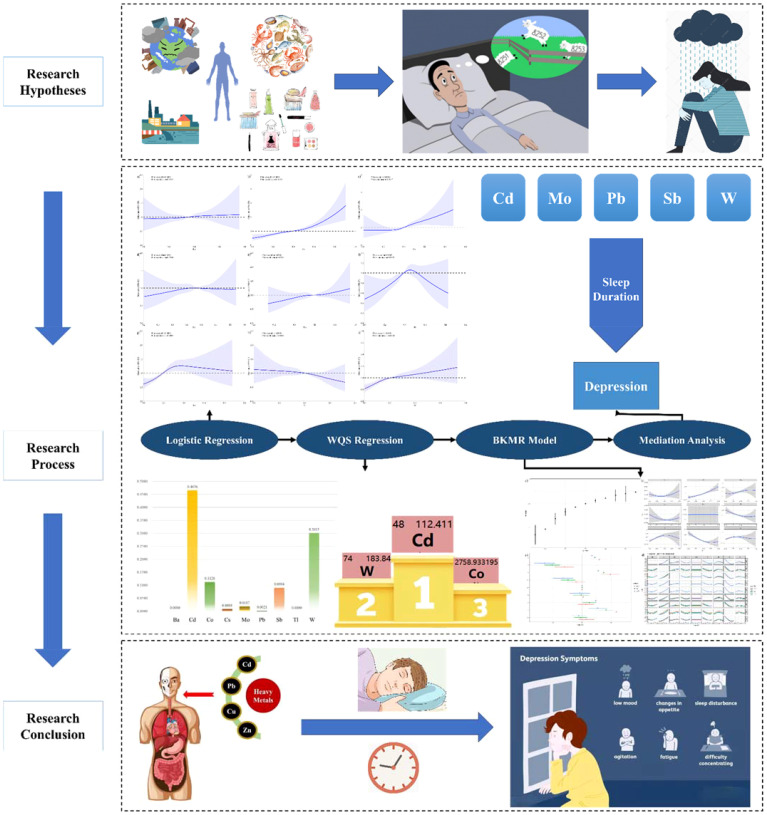

## 1 Introduction

Depression, a complex mental disorder, involves persistent sadness and disinterest, affecting daily functioning and increasing the risk of chronic diseases and economic losses (1). It, projected to become the leading global health burden by 2030 (2), affects millions and significantly impacts mortality in specific groups (3) while straining health budgets (4). Given its escalating prevalence, understanding its etiology is crucial (5). Research indicates a potential link between depression and exposure to heavy metals, which can enter the body through various routes and accumulate in organs, leading to damage in the endocrine, cardiovascular, and nervous systems at high levels (6–8). These metals may also induce oxidative stress, affecting neurotransmitter activity and neuron health, ultimately impacting the central nervous system (9). A cohort study confirmed that urinary antimony was positively related to depressive symptoms, especially in female (10). However, there is no study focusing on the association between heavy metal mixture and depression.

Additionally, there is a well-established connection between sleep duration and depression risk, with both insufficient and excessive sleep linked to greater health issues (11, 12). Specific heavy metals, like manganese, are associated with sleep disturbances. Manganese accumulation in the brain’s basal ganglia can disrupt neuronal activity and alter sleep patterns, as demonstrated by changes in sleep stages in animal studies related to manganese toxicity (13, 14). Notably, individuals with obstructive sleep apnea have been observed to have higher manganese levels than healthy controls (15).

Despite the recognized impact of heavy metals on depression, research exploring the simultaneous exposure to various heavy metals and its effects on depression remains sparse. Moreover, while the relationship between sleep duration and depression is understood, systematic studies examining how sleep patterns mediate the relationship between heavy metal exposure and the development of depression are lacking. This study aims to explore these relationships using data from a large-scale cross-sectional study drawn from national health and nutrition examination survey (NHANES).

## 2 Methodology

### 2.1 Study population

The National Health and Nutrition Examination Survey (NHANES), managed by the CDC’s National Center for Health Statistics, systematically collects health and nutrition data from U.S. civilians using a stratified, four-stage sampling process. Data are gathered every two years, segmented by demographic factors like age, sex, race/ethnicity, and socioeconomic status. After obtaining informed consent, participants complete questionnaires at home and undergo standardized exams at mobile centers. The NHANES database provides free access to anonymized health data. For our study, we analyzed data from eight cycles covering 2005-2020, ensuring a diverse sample across various U.S. regions.

### 2.2 Research design

Informed consent was obtained from all participants. NCHS Ethics Review Board (ERB) Approval Protocol #2011-17 and Protocol #2005-06.

**Figure 1:**
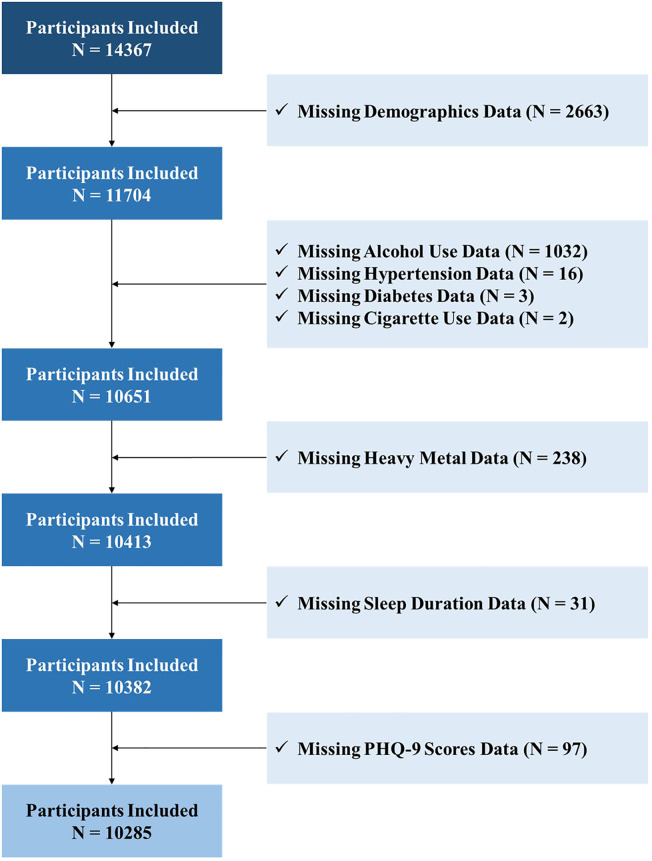
Study Flowchart.

Our initial sample comprised 14,367 participants with complete NHANES data from 2004 to 2018. Due to missing information, 4,082 were excluded, leaving 10,285 individuals for the final analysis (**Figure 1**).

### 2.3 Measurements

For heavy metals, the method involves using mass spectrometry with an inductively coupled plasma (ICP) ionization source to analyze various metals in urine. Samples are diluted, nebulized into droplets, and carried by argon to the ICP. Ions are directed through a focusing area, a dynamic reaction cell (DRC), and a quadrupole mass filter, allowing for sequential detection and isotope identification of each element (16, 17). All data below the detection limit were included in the study of this paper.

As for the recognition of the depression symptoms, The Patient Health Questionnaire (PHQ-9), consisting of nine items, assessed depression by measuring symptom frequency over the past two weeks, plus an extra question evaluating the symptoms’ overall effect. Responses ranged from “not at all” to “nearly every day,” scored from 0 to 3 (18–20). A PHQ-9 score of 10 or below indicated no depression symptoms, while scores above 10 suggested depression symptoms (18).

As for the measurement of sleep duration, sleep duration was measured by the question “How much sleep do you get (hours)?” (2005–2016 cycle) or the question “Number of hours usually sleep on weekdays or workdays.” (2017–2020 cycle)

### 2.4 Statistical analysis

We conducted statistical analysis on depression-related characteristics using Kruskal-Wallis and Fisher’s exact tests for continuous and categorical variables, respectively. Heavy metal concentrations were normalized via log-transformation, and correlations were analyzed using Pearson’s correlation. Logistic regression assessed the impact of heavy metals on depression, with adjustments for demographics and health behaviors.

We applied Weighted Quantile Sum (WQS) regression to study both individual and combined effects of metal mixtures, followed by Bayesian Kernel Machine Regression (BKMR) to examine comprehensive impacts at various exposure levels. Relationships between heavy metal exposure and sleep duration were also explored through linear regression and mediation analysis, employing nonparametric bootstrapping.

Our study incorporated various clinically relevant covariates based on previous findings (21, 22), including age, gender, race/ethnicity, educational level, marital status, family poverty income ratio (PIR), alcohol use, smoking status, diabetes, and hypertension.

NHANES categorizes race/ethnicity into groups such as Mexican American, other Hispanic, non-Hispanic white, non-Hispanic black, and other races (including non-Hispanic Asians and multi-racial individuals). Education is classified from below ninth grade to college graduate or higher, and marital status ranges from married to undisclosed. PIR evaluates annual income relative to poverty thresholds by family size.

Alcohol consumption was determined by annual drinking frequency from 2005 to 2016 and lifetime usage from 2017 to 2020. Smoking was assessed by a history of smoking 100 cigarettes or more. Diabetes and hypertension were self-reported and verified by healthcare providers.

All analyses were conducted in R software, considering results with P < 0.05 as statistically significant (23, 24).

## 3 Results

### 3.1 Baseline data characteristics

**Table 1** illustrates the demographics of the 10,285 participants from the NHANES 2005–2020 dataset. Of these, 705 individuals (percentage: 6.85%) were identified with depression. Significant differences in gender, education, marital status, PIR, diabetes mellitus, hypertension, and smoking habits were observed between those with and without depression (all P < 0.05).

**Table 1:** Characteristics of participants.

	Non-depression		Depression		P-value
	n (Mean)	% (SD)	n (Mean)	% (SD)	
Population	9580	93.15	705	6.85	
Gender					0.0000
Male	4821	50.32	256	36.31	
Female	4759	49.68	449	63.69	
Age	50.3541	17.6692	49.7957	16.4424	0.4160
Race and Ethnicity					0.0053
Mexican American	1343	14.02	108	15.32	
Other Hispanic	849	8.86	90	12.77	
Non-Hispanic White	4212	43.97	296	41.99	
Non-Hispanic Black	2119	22.12	145	20.57	
Other Race – Including Multi-Racial	1057	11.03	66	9.36	
Educational Background					0.0000
Less than 9^th^ grade	835	8.72	104	14.75	
9-11^th^ grade (Includes 12^th^ grade with no diploma)	1213	12.66	133	18.87	
High school graduate/GED or equivalent	2201	22.97	180	25.53	
Some college or AA degree	2919	30.47	221	31.35	
College graduate or above	2412	25.18	67	9.50	
Marital Status					0.0000
Married or Living with Partner	5592	58.37	282	40.00	
Widowed or Divorced or Separated	2173	22.68	250	35.46	
Never married	1815	18.95	173	24.54	
PIR	2.6523	1.6170	1.7201	1.3696	0.0000
Alcohol Use					0.3316
Yes	7229	75.46	544	77.16	
No	2351	24.54	161	22.84	
Hypertension					0.0000
Yes	3437	35.88	346	49.08	
No	6143	64.12	359	50.92	
Diabetes					0.0000
Yes	1202	12.55	159	22.55	
No	8378	87.45	546	77.45	
Cigarette Use					0.0000
Yes	4187	43.71	415	58.87	
No	5393	56.29	290	41.13	
**Sleep Duration**	7.1231	1.4642	6.6184	2.0570	**0.0000**
Heavy Metals					
Ba	0.3537	0.1118	0.3576	0.1113	0.3785
Cd	0.3837	0.1773	0.4343	0.1780	**0.0000**
Co	0.3930	0.1098	0.4105	0.1127	**0.0000**
Cs	0.5621	0.0924	0.5638	0.0904	0.6342
Mo	0.5809	0.1289	0.5889	0.1249	0.1103
Pb	0.3837	0.1183	0.3946	0.1097	**0.0177**
Sb	0.1986	0.1403	0.2280	0.1371	**0.0000**
Tl	0.3954	0.1123	0.3872	0.1114	0.0603
W	0.2050	0.1307	0.2311	0.1279	**0.0000**

The bold value means that P<0.05.

**Supplementary Figure 1** displayed Pearson’s correlation coefficients for the heavy metals’ concentrations. Correlation coefficients for Cs and Tl, Mo and W, and Cs and Mo were notably high (value of correlation: 0.77, 0.60, and 0.60), respectively, whereas correlations among other heavy metals generally ranged between 0.40 and 0.60.

### 3.2 Association between heavy metals and depression

In the logistic regression analyses shown in **Table 2**, significant associations were observed between depression and several heavy metals: Cd (OR (95%CI) = 5.0711 (3.2718 ~ 7.8600)), Co (OR (95%CI) = 4.1443 (2.0951 ~ 8.1978)), Pb (OR (95%CI) = 2.1941 (1.1460 ~ 4.2007)), Sb (OR (95%CI) = 4.0430 (2.4264 ~ 6.7365)), Tl (OR (95%CI) = 0.5245 (0.2675 ~ 1.0284)), and W (OR (95%CI) = 4.4202 (2.5021 ~ 7.8088)) (OR, odd ratio). However, the significant effects of Pb and Co were diminished after controlling for confounders (P > 0.05).

**Table 2:** Results of a single and multiple logistic regression analysis of the correlation between heavy metal exposure and the prevalence of depression.

Variables	Single-factor Logstic Regression					Multifactor Logstic Regression Model I					Multifactor Logstic Regression Model II				
	β	S.E	Z	*P*	OR (95%CI)	β	S.E	Z	*P*	OR (95%CI)	β	S.E	Z	*P*	OR (95%CI)
Ba	0.3076	0.3492	0.8808	0.3784	1.3601 (0.6860 ~ 2.6968)	0.005	0.4112	0.0122	0.9903	1.0050 (0.4489 ~ 2.2500)	-0.021	0.4354	-0.048	0.9614	0.9792 (0.4171 ~ 2.2987)
Cd	1.6236	0.2236	7.2614	**<0.0001**	5.0711 (3.2718 ~ 7.8600)	1.9973	0.2831	7.0554	**<0.0001**	7.3694 (4.2312 ~ 12.8351)	1.6958	0.3107	5.4579	**<.0001**	5.4510 (2.9649 ~ 10.0219)
Co	1.4217	0.348	4.085	**<0.0001**	4.1443 (2.0951 ~ 8.1978)	1.2523	0.4465	2.8047	**0.005**	3.4985 (1.4582 ~ 8.3938)	0.4766	0.4849	0.9829	0.3256	1.6106 (0.6227 ~ 4.1657)
Cs	0.2021	0.4246	0.4759	0.6342	1.2239 (0.5325 ~ 2.8130)	-0.426	0.7706	-0.553	0.5801	0.6529 (0.1442 ~ 2.9567)	0.6202	0.7966	0.7786	0.4362	1.8593 (0.3902 ~ 8.8586)
Mo	0.4893	0.3065	1.5968	0.1103	1.6312 (0.8947 ~ 2.9742)	-0.82	0.4495	-1.825	0.0681	0.4404 (0.1825 ~ 1.0628)	-0.831	0.4639	-1.792	0.0731	0.4355 (0.1754 ~ 1.0811)
Pb	0.7858	0.3314	2.3711	**0.0177**	2.1941 (1.1460 ~ 4.2007)	-1.175	0.4769	-2.464	**0.0138**	0.3089 (0.1213 ~ 0.7865)	-0.758	0.5091	-1.488	0.1368	0.4688 (0.1728 ~ 1.2717)
Sb	1.397	0.2605	5.3628	**<0.0001**	4.0430 (2.4264 ~ 6.7365)	1.1589	0.3286	3.527	**0.0004**	3.1866 (1.6735 ~ 6.0676)	0.8512	0.3534	2.4085	**0.016**	2.3425 (1.1718 ~ 4.6828)
Tl	-0.645	0.3436	-1.879	0.0603	0.5245 (0.2675 ~ 1.0284)	-2.503	0.5674	-4.411	**<0.0001**	0.0818 (0.0269 ~ 0.2489)	-2.112	0.6053	-3.488	**0.0005**	0.1210 (0.0370 ~ 0.3964)
W	1.4862	0.2903	5.1186	**<0.0001**	4.4202 (2.5021 ~ 7.8088)	1.494	0.3756	3.9782	**<0.0001**	4.4550 (2.1339 ~ 9.3007)	1.3988	0.385	3.6333	**0.0003**	4.0503 (1.9045 ~ 8.6135)
Ba						0.2344	0.4349	0.5389	0.5899	1.2642 (0.5390 ~ 2.9650)	0.2513	0.4398	0.5715	0.5677	1.2857 (0.5430 ~ 3.0444)
Cd						1.2031	0.3242	3.7109	**0.0002**	3.3304 (1.7641 ~ 6.2872)	1.1376	0.327	3.479	**0.0005**	3.1192 (1.6433 ~ 5.9207)
Co						0.4856	0.4859	0.9993	0.3176	1.6251 (0.6270 ~ 4.2120)	0.5037	0.4904	1.0272	0.3043	1.6549 (0.6329 ~ 4.3267)
Cs						0.6021	0.7984	0.7542	0.4507	1.8260 (0.3819 ~ 8.7308)	0.8013	0.8013	1.000	0.3173	2.2285 (0.4634 ~ 10.7179)
Mo						-0.641	0.4734	-1.353	0.1761	0.5270 (0.2084 ~ 1.3330)	-0.643	0.4775	-1.346	0.1783	0.5259 (0.2063 ~ 1.3407)
Pb						-0.293	0.5153	-0.568	0.5699	0.7461 (0.2717 ~ 2.0487)	-0.533	0.5214	-1.022	0.3070	0.5871 (0.2113 ~ 1.6311)
Sb						0.7889	0.3604	2.1886	**0.0286**	2.2009 (1.0859 ~ 4.4607)	0.7714	0.3623	2.1291	**0.0332**	2.1629 (1.0632 ~ 4.3999)
Tl						-1.95	0.6032	-3.233	**0.0012**	0.1423 (0.0436 ~ 0.4640)	-1.898	0.6068	-3.128	**0.0018**	0.1499 (0.0456 ~ 0.4923)
W						1.1064	0.3937	2.8099	**0.0050**	3.0234 (1.3975 ~ 6.5412)	1.1249	0.3948	2.8492	**0.0044**	3.0800 (1.4206 ~ 6.6778)

Model I was unadjusted, Model II was adjusted for age, gender, race and ethnicity, educational background, marital status and PIR, Model III was adjusted for age, gender, race and ethnicity, educational background, marital status, PIR, alcohol use status, hypertension, diabetes and cigarette use status, Model IV was adjusted for age, gender, race and ethnicity, educational background, marital status, PIR, alcohol use status, hypertension, diabetes, cigarette use status and sleep duration. The bold value means that P<0.05.

**Figure 2:**
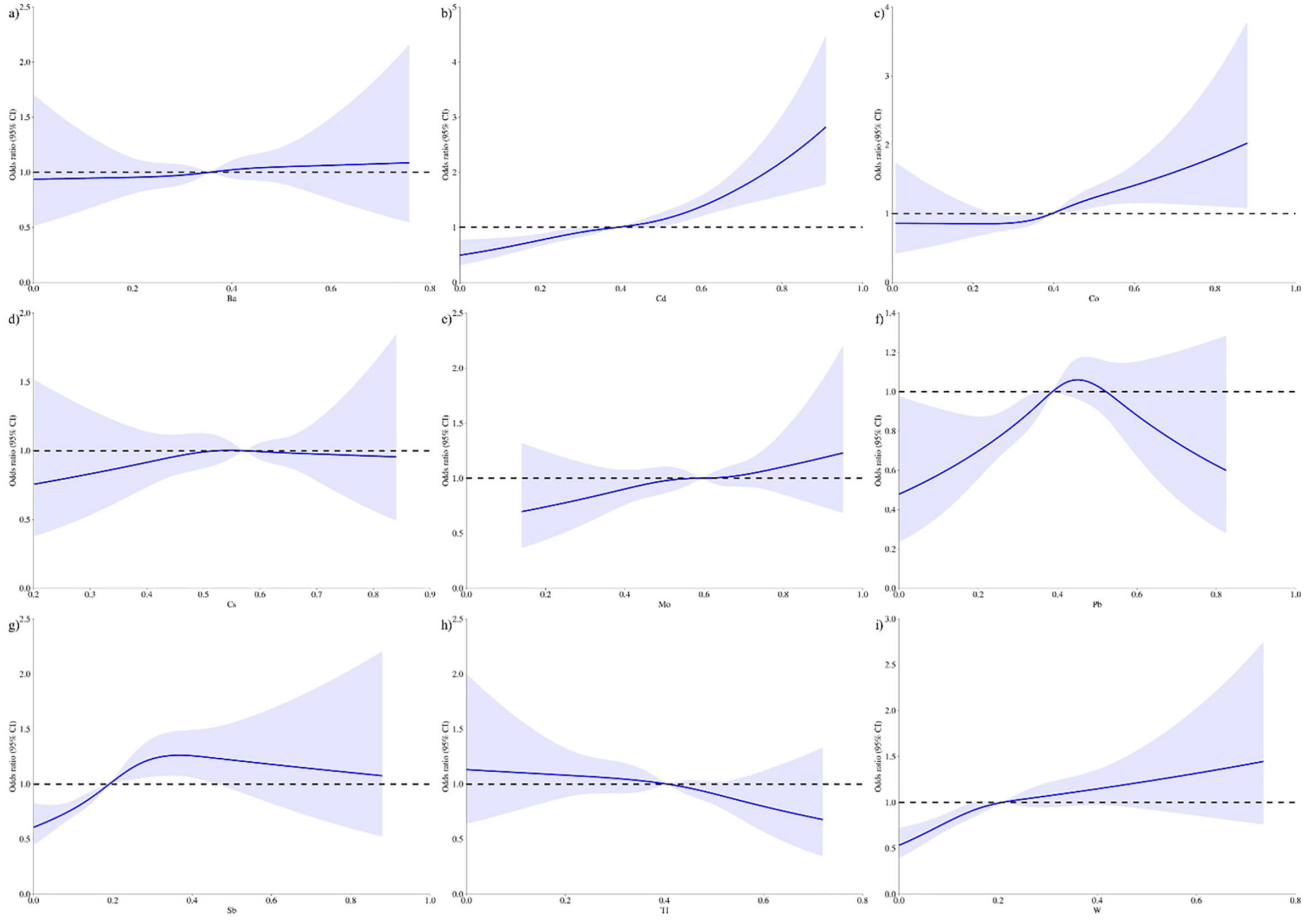
Restricted cubic spline curve describing the dose-response relationship between heavy metal exposure and depression incidence (Unadjusted). : **(A)** Ba, P = 0.8434; **(B)** Cd, P<0.001; **(C)** Co, P = 0.0004; **(D)** Cs, P = 0.8783; **(E)** Mo, P = 0.4286; **(F)** Pb, P = 0.0063; **(G)** Sb, P<0.001; **(H)** Tl, P = 0.2696; **(I)** Ba, P<0.001.

The restricted cubic splines model (RCS) is a nonlinear regression model that uses segmented cubic polynomials to fit the data. RCS depicted an increase in depression prevalence with higher exposure to Ba, Cd, Co, Mo, and W (**Figures 2A–C, E, I**). Conversely, an inverted U-shaped relationship was noted between depression prevalence and Cs, Pb, Sb (**Figures 2D, F, G**). Tl showed a negative correlation with depression prevalence that persisted even after adjustment (**Figure 2H**; **Supplementary Figure 2**).

### 3.3 Subgroup analysis

The correlations between exposure to five heavy metals and both the prevalence and incidence of depression were consistently positive across all analyzed subgroups (**Supplementary Figure 3**).

In subgroup analyses stratified by factors such as drinking and smoking habits, diabetes, and hypertension, no significant interactions were found for Cd, Co, Pb, Sb, and W as they all showed P-values greater than 0.05 (**Supplementary Table 1**). However, gender differences were noted where females, compared to males, showed a higher likelihood of depression with excessive exposure to these five metals. Additionally, in the education subgroup analysis, individuals with higher education levels exhibited an increased risk of depression when exposed to higher levels of Cd and Co.

### 3.4 WQS regression model to assess the association between mixed heavy metals and depression

The WQS model was used to assess the collective impact of nine heavy metals on depression prevalence, as detailed in **Supplementary Table 2**. The results showed a positive association, with the WQS index revealing significant odds ratios (Unadjusted model: OR=0.4624, 95% CI 0.3159–0.6088, P < 0.001; Model I: OR=1.4842, 95% CI 1.2766–1.7254, P < 0.001). Among the metals, Cd was assigned the highest weight of 0.4654, indicating the most substantial influence on depression risk, and this remained the highest even after adjustment for covariates.

### 3.5 BKMR model to assess the association between mixed heavy metals and depression

**Figure 3:**
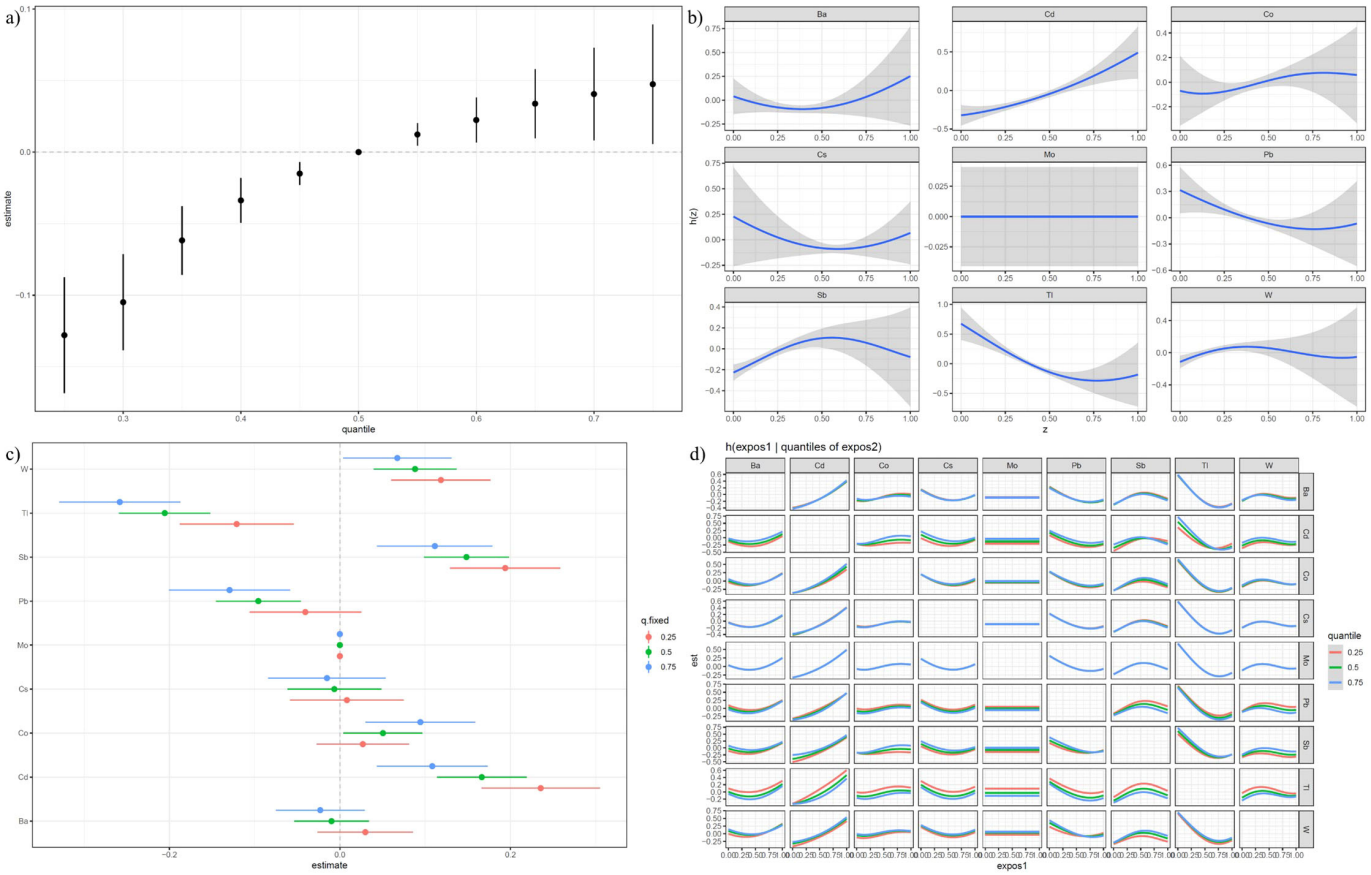
Result of BKMR Model (Unadjusted). **(A)** is overall effect of heavy metals mixtures on the prevalence of depression in BKMR model where all heavy metals at specific percentiles were compared to their 50th percentile. **(B)** is univariate exposure–response function between each heavy metal and the prevalence of depression when the other heavy metals were fixed at 50th percentiles. **(C)** is single exposure-response functions for each heavy metal and prevalence of depression when a single heavy metal was at the 75th compared with the 50th percentile and the concentrations of all the other heavy metals were fixed at either the 25th, 50th, 75th percentile in the BKMR model. **(D)** is bivariate exposure-response functions for each heavy metal and the prevalence of depression when one heavy metal was fixed at 25th, 50th, 75th percentiles and other heavy metals were fixed at the median in the BKMR model.

In the BKMR model without adjustments, the risk of depression increased when exposed to a mixture of heavy metals above the 50th percentile, as shown in **Figure 3A**. In the BKMR model, the PIP value represents the posterior probability of the effect of the variable on the response variable. **Supplementary Table 3** summarized the Posterior Inclusion Probabilities (PIP) indicating most heavy metals had a high PIP linked to depression prevalence. **Figure 3B** illustrated various correlations: a positive correlation with Cd levels, a U-shaped correlation with Ba, Cs, and Tl levels, and an inverted U-shaped correlation with Sb and W levels in depression risk when other metal concentrations were at their median.

Furthermore, **Figure 3C** showed the impact of heavy metals on depression: W, Sb, Co, and Cd had positive effects, whereas Tl and Pb had negative effects when controlled at the 25th, 50th, and 75th percentiles. **Figure 3D** indicated no significant interactions among metal concentrations from the 25th to 75th percentiles.

After covariate adjustment, the pattern of increased depression risk for exposures above the 50th percentile persisted (**Supplementary Figure 4A**). Specific metals showed differing relationships with depression risk when other metals were held at the median: Mo had a negative correlation, while Cs showed a U-shaped and Ba an inverted U-shaped correlation (**Supplementary Figure 4B**). The trends in the effects of metal exposure on depression incidence remained stable across the 25th, 50th, and 75th percentiles (**Supplementary Figure 4C**), with no interactions observed between these concentrations (**Supplementary Figure 4D**).

### 3.6 Mediating role of sleep duration in the association between heavy metals and prevalence of depression

Concentrations of Cs (Estimate (95%CI) = 0.829 (0.255, 1.402), P = 0.0046), Pb (Estimate (95%CI) = -0.597 (-0.949, -0.245), P = 0.0009), and Sb (Estimate (95%CI) =-0.625 (-0.889, -0.361), P = 0.0000) were notably linked to sleep duration, a relationship that persisted even after adjusting for various covariates. Additionally, Cd’s association with sleep duration became significant with covariate adjustments.

Moreover, sleep duration significantly correlated with the prevalence of depression, presenting an odds ratio (OR) of 0.80 (95% CI: 0.76 – 0.84, P < 0.001). This association remained robust after demographic adjustments (OR = 0.81 (0.78 – 0.85)) and further adjustments for all covariates (OR = 0.82 (0.78 – 0.86), P < 0.001).

**Figure 4:**
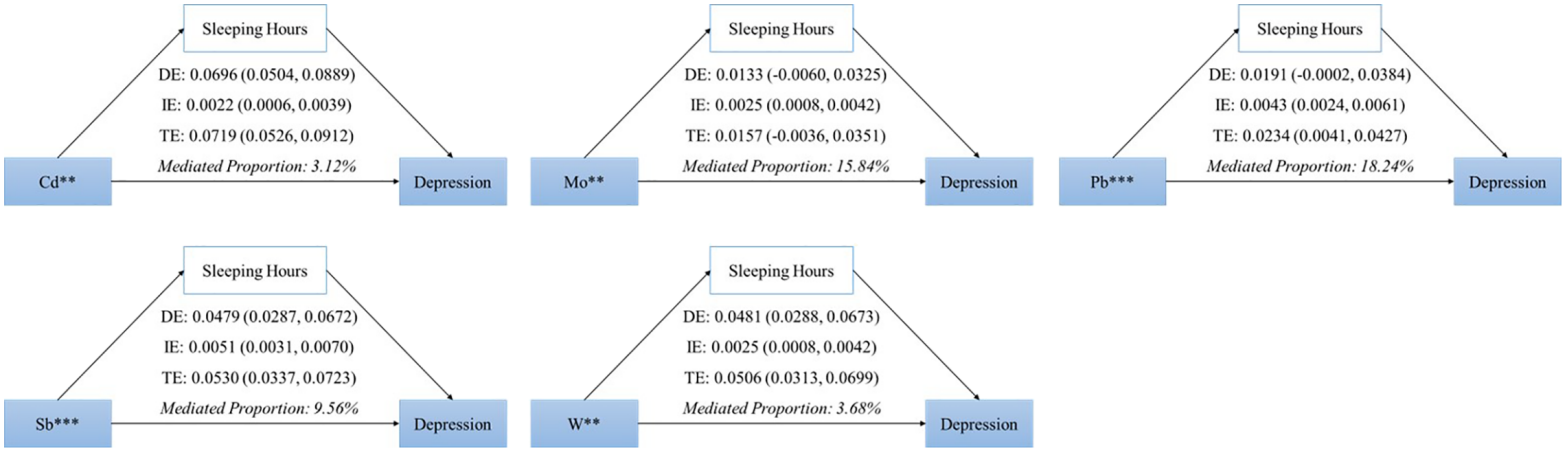
Results of mediation analyses on the association between heavy metal exposure and depression risk mediated by sleep duration (Unadjusted). ** P<0.01, *** P<0.001.

According to Figure 5, sleep duration acted as a mediator in the effects of Cd, Mo, Pb, Sb, and W on depression, contributing mediator shares of 2.55%, 11.46%, 16.60%, 7.99%, and 3.68%, respectively. Post-adjustment for covariates, these associations remained significant, with increased mediator shares reported in the model (4.77%, 13.00%, 20.93%, 14.01%, 6.41%) as shown in **Supplementary Table 4** and **Figure 4**.

## 4 Discussion

Depression is characterized by a significant loss of energy, persistent sadness, sleep disturbances, and anhedonia. A worldwide survey in 17 countries found that 1 in 20 people experience depression. Predictions suggest that by 2020, depression will contribute to a 5.7% rise in the global disease burden, ranking it second only to ischemic heart disease (25).

Recent studies have linked heavy metals to a range of health issues, including obesity, metabolic syndrome, and hypertension (26, 27). Concerns about heavy metal contamination are growing globally (28). Research into how individual heavy metals affect different levels of depression is expanding. For example, higher manganese (Mn) levels have been tied to postnatal depression (29), and studies have noted a connection between elevated lead and cadmium levels in the blood and depression (30). There is an increasing focus on investigating how cadmium (Cd) in the blood may relate to depression, highlighting the broader implications of heavy metal exposure on mental health (31).

Previous studies have not considered the combined effects of multiple metals, leaving it unclear if mixed heavy metal exposure is linked to depression prevalence. Our research using WQS and BKMR models showed that exposure to multiple heavy metals significantly raises the risk of depression, with cadmium (Cd) being a key contributor. These results indicate that high levels of mixed heavy metals, especially Cd, are associated with an increased risk of depression.

There is increasing evidence that the length of sleep significantly affects the likelihood of developing depression. Previous research has mostly examined how a lack of sufficient sleep correlates with a greater risk of depression (32, 33). While the negative impacts of inadequate sleep are well established, the potential risks associated with oversleeping have received less scrutiny. Some studies indicate that both insufficient and excessive sleep durations may contribute to a higher risk of depression (34), a finding our research supports. However, there is disagreement among some experts who believe that extended sleep does not increase the risk of depression (35, 36). These variations in research outcomes may be due to different methods of adjusting for potential confounders, diverse definitions of what constitutes long or short sleep, and the age groups of the participants studied.

Excessive sleep may elevate depression risk by disrupting sleep cycles, leading to increased wakefulness and reduced energy (37, 38). It can also decrease physical activity, impairing brain function and reducing factors that prevent depression such as endorphin release and stress diversion (39). Moreover, individuals who sleep excessively often experience heightened vulnerability to mood and anxiety disorders, and increased stress levels, potentially contributing to depression (40). Additionally, long sleep durations might reflect underlying stress, influencing the link between excessive sleep and depression (41).

Recent studies have shown a connection between longer sleep durations and increased exposure to heavy metals. Research indicates that elevated zinc levels or higher selenium intake can either optimize or prolong sleep in American adults (42), while copper levels do not significantly impact sleep duration (42). Additionally, data from the National Health and Nutrition Examination Survey (NHANES) from 2005 to 2008 suggest that low exposure to antimony is associated with poor sleep health, including shorter sleep duration, increased sleep onset latency, obstructive sleep apnea (OSA), and greater daytime sleepiness (43).

Regarding these common mechanisms, it can be supposed that heavy metal can induce the depression, where sleep duration plays a mediating role in this association, which was confirmed by our study.

The subgroup analysis indicated that the connection between exposure to heavy metals and depression varies by gender, with women at higher risk due to biological variations such as hormonal and metabolic differences (44), along with greater psychosocial and environmental challenges (45, 46). Furthermore, individuals with depression may have higher susceptibility to tobacco, a significant source of heavy metals, as evidenced by elevated urinary metabolites in smokers (46, 47). Tobacco uses itself is a known depressive risk factor (48). These insights underscore the necessity of accounting for gender and socio-demographic factors in environmental health studies to devise precise preventive strategies.

Our study presented multiple strengths.

1. It was pioneering in examining the association between mixed heavy metal exposure and depression prevalence.
2. We employed a variety of statistical techniques and made adjustments for potential confounders, thereby enhancing the validity and reliability of our results.
3. The data used in this study came from a large, population-based database with stringent quality controls in place.

However, there were also notable limitations.

1. Due to the cross-sectional nature of our study, it was not possible to infer causality between heavy metal exposure and depression prevalence, highlight the necessity of the perspective randomized control trials in the future.
2. The NHANES database does not include information on certain uncontrollable factors, such as exposure to wastewater and cosmetics, which could influence the accuracy of our findings.
3. The failure to consider cumulative exposure to heavy metals might have affected our conclusions.

## 5 Conclusion

In summary, our findings show a clear link between exposure to mixed heavy metals and increased depression rates, with cadmium playing a significant role in this association. Additionally, sleep duration serves as a mediator that connects heavy metal exposure to depression prevalence. Future research should focus on further exploring this relationship through prospective studies.

## Data Availability

The raw data supporting the conclusions of this article will be made available by the authors, without undue reservation.
