# Supplementary material for: Sleep duration mediates the association between heavy metals and the prevalence of depression: an integrated approach from the NHANES (2005–2020)
Source: Front Psychiatry. 2024 Sep 2;15:1455896. doi: 10.3389/fpsyt.2024.1455896 (PMC11404323; doi:10.3389/fpsyt.2024.1455896)
Supplement: Supplementary file 1 [file DataSheet1.docx]

Table S1 Association between Mixed Heavy Metal and Total BMD Assessed by Multiple Linear Regression Analysis (Adjusted for age, gender, race and ethnicity, educational background, marital status, drinking status, hypertension, diabetes status and smoking status)

| Variables | Multiple Linear Regression Analysis | | | | |
| --- | --- | --- | --- | --- | --- |
|  | b | S.E | t | *P* | β (95%CI) |
| Ba | -0.0628 | 0.0293 | -2.1429 | **0.0322** | -0.0628 (-0.1203 ~ -0.0054) |
| Cd | -0.0114 | 0.0193 | -0.591 | 0.5546 | -0.0114 (-0.0492 ~ 0.0264) |
| Co | 0.0402 | 0.0276 | 1.4559 | 0.1456 | 0.0402 (-0.0139 ~ 0.0943) |
| Cs | 0.1446 | 0.0492 | 2.9399 | **0.0033** | 0.1446 (0.0482 ~ 0.2410) |
| Mo | -0.0212 | 0.0266 | -0.7982 | 0.4248 | -0.0212 (-0.0733 ~ 0.0309) |
| Mn | 0.0652 | 0.0274 | 2.3771 | **0.0175** | 0.0652 (0.0114 ~ 0.1190) |
| Pb | -0.1282 | 0.031 | -4.1402 | **<.0001** | -0.1282 (-0.1888 ~ -0.0675) |
| Sb | 0.0327 | 0.0205 | 1.5949 | 0.1109 | 0.0327 (-0.0075 ~ 0.0730) |
| Sn | 0.0003 | 0.0192 | 0.0136 | 0.9892 | 0.0003 (-0.0374 ~ 0.0380) |
| Sr | 0.0057 | 0.0316 | 0.1806 | 0.8567 | 0.0057 (-0.0562 ~ 0.0677) |
| Tl | 0.0376 | 0.0293 | 1.2843 | 0.1992 | 0.0376 (-0.0198 ~ 0.0950) |
| W | 0.0177 | 0.0252 | 0.7027 | 0.4823 | 0.0177 (-0.0317 ~ 0.0671) |
| U | -0.0236 | 0.0203 | -1.1644 | 0.2444 | -0.0236 (-0.0633 ~ 0.0161) |

Table S2 Single Effect of Basic nutrients Intake on the Association between Cd and Total BMD

| Independent Variable | Intermediary Variable | Predictor Variable | Dierct Effects β (95% CI) | | |  | Indierct Effects β (95% CI) | | |  | Total Effects β (95% CI) | | | Mediated Proportion | P-value |
| --- | --- | --- | --- | --- | --- | --- | --- | --- | --- | --- | --- | --- | --- | --- | --- |
|  |  |  | Estimate | CI Lower | CI Upper |  | Estimate | CI Lower | CI Upper |  | Estimate | CI Lower | CI Upper |  |  |
| Cd | Calorie | Total BMD | -0.0064 | -0.0477 | 0.0350 |  | -0.0061 | -0.0108 | -0.0014 |  | -0.0125 | -0.0539 | 0.0290 | 0.4897 | 0.0109 |
| Cd | Protein | Total BMD | -0.0020 | -0.0434 | 0.0393 |  | -0.0104 | -0.0165 | -0.0044 |  | -0.0125 | -0.0539 | 0.0290 | 0.8366 | 0.0007 |
| Cd | Fat | Total BMD | -0.0057 | -0.0470 | 0.0357 |  | -0.0068 | -0.0119 | -0.0017 |  | -0.0125 | -0.0539 | 0.0290 | 0.5453 | 0.0094 |

Table S3 Association between Mixed Heavy Metal and BMD in Specific Areas Assessed by Multiple Linear Regression Analysis (Adjusted for age, gender, race and ethnicity, educational background, marital status, drinking status, hypertension, diabetes status and smoking status)

| Variables | Multiple Linear Regression Analysis | | | | |
| --- | --- | --- | --- | --- | --- |
|  | b | S.E | t | *P* | β (95%CI) |
| **Head Bone** | | | | | |
| Ba | -0.0627 | 0.0367 | -1.7075 | 0.0879 | -0.0627 (-0.1346 ~ 0.0093) |
| Cd | -0.0004 | 0.0242 | -0.0185 | **0.9852** | -0.0004 (-0.0478 ~ 0.0469) |
| Co | 0.0845 | 0.0346 | 2.4445 | **0.0146** | 0.0845 (0.0167 ~ 0.1522) |
| Cs | 0.0729 | 0.0616 | 1.1837 | 0.2366 | 0.0729 (-0.0478 ~ 0.1937) |
| Mo | -0.013 | 0.0333 | -0.3898 | **0.6967** | -0.0130 (-0.0782 ~ 0.0522) |
| Mn | 0.0305 | 0.0344 | 0.8878 | 0.3748 | 0.0305 (-0.0368 ~ 0.0979) |
| Pb | -0.1789 | 0.0388 | -4.6143 | **<.0001** | -0.1789 (-0.2549 ~ -0.1029) |
| Sb | 0.0357 | 0.0257 | 1.3869 | 0.1656 | 0.0357 (-0.0147 ~ 0.0860) |
| Sn | 0.029 | 0.0241 | 1.2035 | **0.2289** | 0.0290 (-0.0182 ~ 0.0762) |
| Sr | 0.005 | 0.0396 | 0.1254 | **0.9002** | 0.0050 (-0.0726 ~ 0.0826) |
| Tl | 0.0258 | 0.0367 | 0.7018 | 0.4829 | 0.0258 (-0.0462 ~ 0.0977) |
| W | 0.0175 | 0.0316 | 0.5536 | 0.5799 | 0.0175 (-0.0444 ~ 0.0794) |
| U | -0.0074 | 0.0254 | -0.2919 | 0.7704 | -0.0074 (-0.0572 ~ 0.0424) |
| **Left Arm** | | | | | |
| Ba | 0.0016 | 0.026 | 0.0598 | 0.9523 | 0.0016 (-0.0494 ~ 0.0525) |
| Cd | -0.0239 | 0.0171 | -1.397 | **0.1626** | -0.0239 (-0.0575 ~ 0.0096) |
| Co | 0.0281 | 0.0245 | 1.1485 | **0.2509** | 0.0281 (-0.0199 ~ 0.0761) |
| Cs | 0.0947 | 0.0437 | 2.1696 | **0.0301** | 0.0947 (0.0092 ~ 0.1803) |
| Mo | -0.0121 | 0.0236 | -0.5149 | 0.6067 | -0.0121 (-0.0583 ~ 0.0341) |
| Mn | 0.049 | 0.0243 | 2.0109 | 0.0445 | 0.0490 (0.0012 ~ 0.0967) |
| Pb | -0.0646 | 0.0275 | -2.3524 | **0.0187** | -0.0646 (-0.1185 ~ -0.0108) |
| Sb | 0.0349 | 0.0182 | 1.9171 | **0.0554** | 0.0349 (-0.0008 ~ 0.0706) |
| Sn | 0.0153 | 0.0171 | 0.8981 | **0.3692** | 0.0153 (-0.0181 ~ 0.0488) |
| Sr | -0.0455 | 0.0281 | -1.623 | 0.1047 | -0.0455 (-0.1005 ~ 0.0095) |
| Tl | 0.0389 | 0.026 | 1.4961 | **0.1348** | 0.0389 (-0.0121 ~ 0.0898) |
| W | -0.0033 | 0.0224 | -0.1493 | 0.8814 | -0.0033 (-0.0472 ~ 0.0405) |
| U | 0.0013 | 0.018 | 0.0745 | 0.9406 | 0.0013 (-0.0339 ~ 0.0366) |
| **Left Leg** | | | | | |
| Ba | -0.0335 | 0.0222 | -1.5089 | 0.1315 | -0.0335 (-0.0771 ~ 0.0100) |
| Cd | -0.017 | 0.0146 | -1.1636 | **0.2447** | -0.0170 (-0.0457 ~ 0.0116) |
| Co | 0.0061 | 0.0209 | 0.2915 | **0.7707** | 0.0061 (-0.0349 ~ 0.0471) |
| Cs | 0.1005 | 0.0373 | 2.6941 | **0.0071** | 0.1005 (0.0274 ~ 0.1736) |
| Mo | -0.0075 | 0.0201 | -0.3701 | 0.7114 | -0.0075 (-0.0469 ~ 0.0320) |
| Mn | 0.0582 | 0.0208 | 2.7989 | 0.0052 | 0.0582 (0.0174 ~ 0.0990) |
| Pb | -0.0888 | 0.0235 | -3.7833 | 0.0002 | -0.0888 (-0.1348 ~ -0.0428) |
| Sb | 0.0359 | 0.0156 | 2.31 | **0.021** | 0.0359 (0.0054 ~ 0.0664) |
| Sn | -0.0052 | 0.0146 | -0.3545 | **0.723** | -0.0052 (-0.0337 ~ 0.0234) |
| Sr | 0.0053 | 0.024 | 0.2232 | 0.8234 | 0.0053 (-0.0416 ~ 0.0523) |
| Tl | 0.0415 | 0.0222 | 1.8669 | **0.0621** | 0.0415 (-0.0021 ~ 0.0850) |
| W | 0.0151 | 0.0191 | 0.7902 | **0.4295** | 0.0151 (-0.0224 ~ 0.0526) |
| U | -0.0174 | 0.0154 | -1.1304 | 0.2584 | -0.0174 (-0.0475 ~ 0.0127) |
| **Right Arm** | | | | | |
| Ba | -0.0106 | 0.0272 | -0.3891 | 0.6972 | -0.0106 (-0.0638 ~ 0.0427) |
| Cd | -0.0304 | 0.0179 | -1.6998 | **0.0893** | -0.0304 (-0.0654 ~ 0.0047) |
| Co | 0.0171 | 0.0256 | 0.6674 | **0.5046** | 0.0171 (-0.0331 ~ 0.0672) |
| Cs | 0.0956 | 0.0456 | 2.0973 | **0.0361** | 0.0956 (0.0063 ~ 0.1850) |
| Mo | -0.0211 | 0.0246 | -0.858 | 0.391 | -0.0211 (-0.0694 ~ 0.0271) |
| Mn | 0.0465 | 0.0254 | 1.8303 | 0.0673 | 0.0465 (-0.0033 ~ 0.0964) |
| Pb | -0.0483 | 0.0287 | -1.6831 | **0.0925** | -0.0483 (-0.1045 ~ 0.0079) |
| Sb | 0.032 | 0.019 | 1.6808 | **0.0929** | 0.0320 (-0.0053 ~ 0.0693) |
| Sn | -0.0042 | 0.0178 | -0.2332 | **0.8156** | -0.0042 (-0.0391 ~ 0.0308) |
| Sr | -0.0249 | 0.0293 | -0.8487 | 0.3962 | -0.0249 (-0.0823 ~ 0.0326) |
| Tl | 0.0467 | 0.0271 | 1.719 | **0.0858** | 0.0467 (-0.0065 ~ 0.0999) |
| W | -0.0015 | 0.0234 | -0.0639 | 0.9491 | -0.0015 (-0.0473 ~ 0.0443) |
| U | 0.0068 | 0.0188 | 0.3628 | 0.7168 | 0.0068 (-0.0300 ~ 0.0436) |
| **Right Leg** | | | | | |
| Ba | 0.005 | 0.0247 | 0.2008 | **0.8409** | 0.0050 (-0.0434 ~ 0.0533) |
| Cd | -0.0343 | 0.0162 | -2.1168 | **0.0344** | -0.0343 (-0.0661 ~ -0.0025) |
| Co | 0.0103 | 0.0232 | 0.4448 | **0.6565** | 0.0103 (-0.0352 ~ 0.0558) |
| Cs | 0.128 | 0.0414 | 3.0942 | **0.002** | 0.1280 (0.0469 ~ 0.2091) |
| Mo | 0.006 | 0.0223 | 0.2672 | **0.7893** | 0.0060 (-0.0378 ~ 0.0498) |
| Mn | 0.0443 | 0.0231 | 1.9185 | 0.0552 | 0.0443 (-0.0010 ~ 0.0895) |
| Pb | -0.0851 | 0.026 | -3.268 | 0.0011 | -0.0851 (-0.1361 ~ -0.0341) |
| Sb | 0.0418 | 0.0173 | 2.4222 | **0.0155** | 0.0418 (0.0080 ~ 0.0757) |
| Sn | 0.0007 | 0.0162 | 0.046 | **0.9633** | 0.0007 (-0.0310 ~ 0.0325) |
| Sr | -0.0523 | 0.0266 | -1.9687 | 0.0491 | -0.0523 (-0.1044 ~ -0.0002) |
| Tl | 0.055 | 0.0246 | 2.2306 | **0.0258** | 0.0550 (0.0067 ~ 0.1032) |
| W | 0.0055 | 0.0212 | 0.2602 | **0.7948** | 0.0055 (-0.0360 ~ 0.0471) |
| U | -0.0035 | 0.017 | -0.2078 | 0.8354 | -0.0035 (-0.0370 ~ 0.0299) |
| **Left Rib** | | | | | |
| Ba | -0.0523 | 0.0308 | -1.702 | 0.0889 | -0.0523 (-0.1126 ~ 0.0079) |
| Cd | -0.0201 | 0.0202 | -0.9954 | **0.3197** | -0.0201 (-0.0598 ~ 0.0195) |
| Co | 0.0223 | 0.029 | 0.7704 | **0.4411** | 0.0223 (-0.0344 ~ 0.0791) |
| Cs | 0.1397 | 0.0516 | 2.7061 | 0.0069 | 0.1397 (0.0385 ~ 0.2408) |
| Mo | -0.0478 | 0.0279 | -1.7147 | 0.0865 | -0.0478 (-0.1024 ~ 0.0068) |
| Mn | 0.0383 | 0.0288 | 1.3321 | 0.183 | 0.0383 (-0.0181 ~ 0.0948) |
| Pb | -0.0451 | 0.0325 | -1.3872 | 0.1655 | -0.0451 (-0.1087 ~ 0.0186) |
| Sb | 0.0199 | 0.0215 | 0.9229 | **0.3561** | 0.0199 (-0.0223 ~ 0.0621) |
| Sn | 0.0092 | 0.0202 | 0.454 | 0.6499 | 0.0092 (-0.0304 ~ 0.0487) |
| Sr | 0.0033 | 0.0332 | 0.1 | 0.9203 | 0.0033 (-0.0617 ~ 0.0683) |
| Tl | 0.0291 | 0.0307 | 0.9481 | **0.3432** | 0.0291 (-0.0311 ~ 0.0894) |
| W | 0.0047 | 0.0265 | 0.1771 | **0.8594** | 0.0047 (-0.0472 ~ 0.0565) |
| U | -0.0187 | 0.0213 | -0.8795 | 0.3793 | -0.0187 (-0.0604 ~ 0.0230) |
| **Right Rib** | | | | | |
| Ba | -0.0604 | 0.0306 | -1.9714 | 0.0488 | -0.0604 (-0.1204 ~ -0.0004) |
| Cd | -0.0207 | 0.0202 | -1.0271 | **0.3045** | -0.0207 (-0.0602 ~ 0.0188) |
| Co | 0.0026 | 0.0288 | 0.0899 | **0.9284** | 0.0026 (-0.0539 ~ 0.0591) |
| Cs | 0.135 | 0.0514 | 2.6271 | 0.0087 | 0.1350 (0.0343 ~ 0.2358) |
| Mo | -0.0379 | 0.0278 | -1.3665 | 0.1719 | -0.0379 (-0.0923 ~ 0.0165) |
| Mn | 0.0363 | 0.0287 | 1.2678 | 0.205 | 0.0363 (-0.0198 ~ 0.0925) |
| Pb | -0.0397 | 0.0323 | -1.2282 | 0.2195 | -0.0397 (-0.1031 ~ 0.0237) |
| Sb | 0.0404 | 0.0215 | 1.8825 | **0.0599** | 0.0404 (-0.0017 ~ 0.0824) |
| Sn | 0.0059 | 0.0201 | 0.2921 | 0.7702 | 0.0059 (-0.0335 ~ 0.0453) |
| Sr | -0.0029 | 0.033 | -0.0892 | 0.9289 | -0.0029 (-0.0677 ~ 0.0618) |
| Tl | 0.0515 | 0.0306 | 1.6814 | **0.0928** | 0.0515 (-0.0085 ~ 0.1115) |
| W | -0.0032 | 0.0263 | -0.1208 | **0.9038** | -0.0032 (-0.0548 ~ 0.0485) |
| U | -0.0256 | 0.0212 | -1.2082 | 0.2271 | -0.0256 (-0.0671 ~ 0.0159) |
| **Thoracic Spine** | | | | | |
| Ba | -0.0017 | 0.0301 | -0.0575 | 0.9542 | -0.0017 (-0.0607 ~ 0.0573) |
| Cd | -0.0268 | 0.0198 | -1.3513 | 0.1767 | -0.0268 (-0.0656 ~ 0.0121) |
| Co | 0.0289 | 0.0283 | 1.0186 | **0.3085** | 0.0289 (-0.0267 ~ 0.0844) |
| Cs | 0.1921 | 0.0505 | 3.802 | **0.0001** | 0.1921 (0.0931 ~ 0.2911) |
| Mo | -0.0446 | 0.0273 | -1.6335 | 0.1025 | -0.0446 (-0.0980 ~ 0.0089) |
| Mn | 0.0384 | 0.0282 | 1.3634 | 0.1729 | 0.0384 (-0.0168 ~ 0.0936) |
| Pb | -0.1551 | 0.0318 | -4.8785 | **<.0001** | -0.1551 (-0.2174 ~ -0.0928) |
| Sb | 0.0319 | 0.0211 | 1.5154 | **0.1298** | 0.0319 (-0.0094 ~ 0.0733) |
| Sn | -0.0026 | 0.0198 | -0.1313 | 0.8956 | -0.0026 (-0.0413 ~ 0.0361) |
| Sr | -0.0215 | 0.0325 | -0.663 | 0.5074 | -0.0215 (-0.0851 ~ 0.0421) |
| Tl | 0.0613 | 0.0301 | 2.0366 | **0.0418** | 0.0613 (0.0023 ~ 0.1202) |
| W | 0.0186 | 0.0259 | 0.7185 | 0.4725 | 0.0186 (-0.0321 ~ 0.0694) |
| U | -0.0115 | 0.0208 | -0.5519 | 0.5811 | -0.0115 (-0.0523 ~ 0.0293) |
| **Lumbar Spine** | | | | | |
| Ba | -0.0533 | 0.0316 | -1.6902 | 0.0911 | -0.0533 (-0.1152 ~ 0.0085) |
| Cd | -0.0135 | 0.0208 | -0.6477 | 0.5172 | -0.0135 (-0.0542 ~ 0.0273) |
| Co | 0.0393 | 0.0297 | 1.3241 | 0.1856 | 0.0393 (-0.0189 ~ 0.0976) |
| Cs | 0.1076 | 0.053 | 2.0311 | 0.0424 | 0.1076 (0.0038 ~ 0.2114) |
| Mo | -0.0216 | 0.0286 | -0.7545 | 0.4506 | -0.0216 (-0.0776 ~ 0.0345) |
| Mn | 0.0177 | 0.0295 | 0.5988 | 0.5494 | 0.0177 (-0.0402 ~ 0.0756) |
| Pb | -0.0729 | 0.0333 | -2.1859 | **0.0289** | -0.0729 (-0.1382 ~ -0.0075) |
| Sb | 0.0173 | 0.0221 | 0.7833 | **0.4336** | 0.0173 (-0.0260 ~ 0.0606) |
| Sn | 0.0076 | 0.0207 | 0.3671 | 0.7136 | 0.0076 (-0.0330 ~ 0.0482) |
| Sr | 0.0057 | 0.034 | 0.1688 | 0.866 | 0.0057 (-0.0610 ~ 0.0724) |
| Tl | 0.0098 | 0.0315 | 0.3103 | 0.7563 | 0.0098 (-0.0520 ~ 0.0716) |
| W | 0.019 | 0.0271 | 0.6996 | **0.4842** | 0.0190 (-0.0342 ~ 0.0722) |
| U | -0.0295 | 0.0218 | -1.35 | 0.1772 | -0.0295 (-0.0722 ~ 0.0133) |
| **Pelvis** | | | | | |
| Ba | 0.04 | 0.0297 | 1.347 | 0.1781 | 0.0400 (-0.0182 ~ 0.0983) |
| Cd | -0.0315 | 0.0196 | -1.609 | **0.1078** | -0.0315 (-0.0698 ~ 0.0069) |
| Co | -0.0098 | 0.028 | -0.3495 | **0.7268** | -0.0098 (-0.0646 ~ 0.0451) |
| Cs | 0.1148 | 0.0499 | 2.3 | 0.0215 | 0.1148 (0.0170 ~ 0.2125) |
| Mo | -0.0013 | 0.0269 | -0.0484 | 0.9614 | -0.0013 (-0.0541 ~ 0.0515) |
| Mn | 0.0145 | 0.0278 | 0.521 | 0.6024 | 0.0145 (-0.0400 ~ 0.0690) |
| Pb | -0.101 | 0.0314 | -3.2176 | **0.0013** | -0.1010 (-0.1626 ~ -0.0395) |
| Sb | 0.0168 | 0.0208 | 0.8046 | **0.4211** | 0.0168 (-0.0241 ~ 0.0576) |
| Sn | -0.0328 | 0.0195 | -1.6836 | 0.0924 | -0.0328 (-0.0711 ~ 0.0054) |
| Sr | -0.0136 | 0.0321 | -0.4243 | 0.6714 | -0.0136 (-0.0764 ~ 0.0492) |
| Tl | 0.0795 | 0.0297 | 2.6773 | **0.0075** | 0.0795 (0.0213 ~ 0.1378) |
| W | 0.0295 | 0.0256 | 1.1544 | **0.2485** | 0.0295 (-0.0206 ~ 0.0796) |
| U | -0.0181 | 0.0206 | -0.8808 | 0.3785 | -0.0181 (-0.0584 ~ 0.0222) |
| **Trunk Bone** | | | | | |
| Ba | -0.0333 | 0.0272 | -1.2239 | 0.2211 | -0.0333 (-0.0866 ~ 0.0200) |
| Cd | -0.0265 | 0.0179 | -1.4832 | **0.1382** | -0.0265 (-0.0616 ~ 0.0085) |
| Co | 0.0151 | 0.0256 | 0.588 | **0.5566** | 0.0151 (-0.0351 ~ 0.0652) |
| Cs | 0.1578 | 0.0456 | 3.4578 | **0.0006** | 0.1578 (0.0684 ~ 0.2473) |
| Mo | -0.0212 | 0.0246 | -0.8596 | 0.3901 | -0.0212 (-0.0695 ~ 0.0271) |
| Mn | 0.037 | 0.0255 | 1.4532 | 0.1463 | 0.0370 (-0.0129 ~ 0.0869) |
| Pb | -0.0879 | 0.0287 | -3.0614 | **0.0022** | -0.0879 (-0.1442 ~ -0.0316) |
| Sb | 0.0197 | 0.019 | 1.0332 | **0.3016** | 0.0197 (-0.0176 ~ 0.0570) |
| Sn | -0.0115 | 0.0178 | -0.6432 | 0.5202 | -0.0115 (-0.0465 ~ 0.0235) |
| Sr | 0.0022 | 0.0293 | 0.0759 | 0.9395 | 0.0022 (-0.0553 ~ 0.0597) |
| Tl | 0.0428 | 0.0272 | 1.5759 | **0.1152** | 0.0428 (-0.0104 ~ 0.0961) |
| W | 0.0153 | 0.0234 | 0.6538 | **0.5133** | 0.0153 (-0.0306 ~ 0.0611) |
| U | -0.0267 | 0.0188 | -1.4177 | 0.1564 | -0.0267 (-0.0635 ~ 0.0102) |

Table S4 Single Effect of Basic nutrients Intake on the Association between Cd and BMD in Specific Areas

| Independent Variable | Intermediary Variable | Predictor Variable | Dierct Effects β (95% CI) | | |  | Indierct Effects β (95% CI) | | |  | Total Effects β (95% CI) | | | Mediated Proportion | P-value |
| --- | --- | --- | --- | --- | --- | --- | --- | --- | --- | --- | --- | --- | --- | --- | --- |
|  |  |  | Estimate | CI Lower | CI Upper |  | Estimate | CI Lower | CI Upper |  | Estimate | CI Lower | CI Upper |  |  |
| Cd | Calorie | Head Bone | 0.0566 | 0.0153 | 0.0979 |  | 0.0055 | 0.0011 | 0.0099 |  | 0.0621 | 0.0207 | 0.1035 | 0.0889 | 0.0140 |
| Cd | Protein | Head Bone | 0.0579 | 0.0163 | 0.0994 |  | 0.0043 | 0.0000 | 0.0085 |  | 0.0621 | 0.0207 | 0.1035 | 0.0687 | 0.0498 |
| Cd | Dietary fiber | Head Bone | 0.0556 | 0.0139 | 0.0972 |  | 0.0066 | 0.0010 | 0.0121 |  | 0.0621 | 0.0207 | 0.1035 | 0.1055 | 0.0215 |
| Cd | Calorie | Left Arm | 0.0060 | -0.0346 | 0.0466 |  | -0.0145 | -0.0239 | -0.0050 |  | -0.0085 | -0.0500 | 0.0330 | 1.7055 | 0.0027 |
| Cd | Protein | Left Arm | 0.0116 | -0.0290 | 0.0523 |  | -0.0201 | -0.0299 | -0.0103 |  | -0.0085 | -0.0500 | 0.0330 | 2.3697 | 0.0001 |
| Cd | Carbohydrate | Left Arm | -0.0018 | -0.0429 | 0.0392 |  | -0.0066 | -0.0130 | -0.0003 |  | -0.0085 | -0.0500 | 0.0330 | 0.7834 | 0.0388 |
| Cd | Fat | Left Arm | 0.0038 | -0.0370 | 0.0446 |  | -0.0123 | -0.0207 | -0.0039 |  | -0.0085 | -0.0500 | 0.0330 | 1.4519 | 0.0039 |
| Cd | Calorie | Right Arm | -0.0092 | -0.0498 | 0.0314 |  | -0.0144 | -0.0238 | -0.0050 |  | -0.0236 | -0.0651 | 0.0179 | 0.6101 | 0.0027 |
| Cd | Protein | Right Arm | -0.0033 | -0.0439 | 0.0373 |  | -0.0203 | -0.0302 | -0.0104 |  | -0.0236 | -0.0651 | 0.0179 | 0.8597 | 0.0001 |
| Cd | Carbohydrate | Right Arm | -0.0171 | -0.0582 | 0.0240 |  | -0.0065 | -0.0127 | -0.0003 |  | -0.0236 | -0.0651 | 0.0179 | 0.2767 | 0.0391 |
| Cd | Fat | Right Arm | -0.0115 | -0.0523 | 0.0293 |  | -0.0121 | -0.0204 | -0.0039 |  | -0.0236 | -0.0651 | 0.0179 | 0.5136 | 0.0040 |
| Cd | Calorie | Left Rib | -0.0328 | -0.0741 | 0.0085 |  | -0.0074 | -0.0128 | -0.0020 |  | -0.0402 | -0.0816 | 0.0012 | 0.1845 | 0.0069 |
| Cd | Protein | Left Rib | -0.0296 | -0.0710 | 0.0117 |  | -0.0106 | -0.0167 | -0.0045 |  | -0.0402 | -0.0816 | 0.0012 | 0.2634 | 0.0007 |
| Cd | Fat | Left Rib | -0.0333 | -0.0746 | 0.0080 |  | -0.0069 | -0.0121 | -0.0017 |  | -0.0402 | -0.0816 | 0.0012 | 0.1716 | 0.0090 |
| Cd | Calorie | Right Rib | -0.0337 | -0.0750 | 0.0075 |  | -0.0074 | -0.0128 | -0.0020 |  | -0.0412 | -0.0826 | 0.0003 | 0.1807 | 0.0069 |
| Cd | Protein | Right Rib | -0.0305 | -0.0718 | 0.0108 |  | -0.0107 | -0.0168 | -0.0046 |  | -0.0412 | -0.0826 | 0.0003 | 0.2594 | 0.0006 |
| Cd | Fat | Right Rib | -0.0344 | -0.0757 | 0.0069 |  | -0.0068 | -0.0119 | -0.0017 |  | -0.0412 | -0.0826 | 0.0003 | 0.1639 | 0.0095 |
| Cd | Calorie | Thoracic Spine | 0.0310 | -0.0105 | 0.0725 |  | -0.0045 | -0.0085 | -0.0006 |  | 0.0265 | -0.0150 | 0.0679 | -0.1718 | 0.0241 |
| Cd | Protein | Thoracic Spine | 0.0351 | -0.0063 | 0.0766 |  | -0.0087 | -0.0141 | -0.0032 |  | 0.0265 | -0.0150 | 0.0679 | -0.3271 | 0.0018 |
| Cd | Fat | Thoracic Spine | 0.0324 | -0.0090 | 0.0738 |  | -0.0059 | -0.0106 | -0.0013 |  | 0.0265 | -0.0150 | 0.0679 | -0.2240 | 0.0126 |
| Cd | Dietary fiber | Lumbar Spine | -0.0302 | -0.0719 | 0.0115 |  | 0.0059 | 0.0004 | 0.0115 |  | -0.0243 | -0.0657 | 0.0172 | -0.2448 | 0.0349 |
| Cd | Calorie | Pelvis | -0.0233 | -0.0648 | 0.0182 |  | -0.0040 | -0.0078 | -0.0003 |  | -0.0274 | -0.0688 | 0.0141 | 0.1476 | 0.0339 |
| Cd | Protein | Pelvis | -0.0221 | -0.0637 | 0.0195 |  | -0.0052 | -0.0097 | -0.0008 |  | -0.0274 | -0.0688 | 0.0141 | 0.1917 | 0.0219 |
| Cd | Fat | Pelvis | -0.0227 | -0.0641 | 0.0188 |  | -0.0047 | -0.0087 | -0.0007 |  | -0.0274 | -0.0688 | 0.0141 | 0.1717 | 0.0224 |
| Cd | Calorie | Trunk Bone | -0.0378 | -0.0791 | 0.0036 |  | -0.0060 | -0.0106 | -0.0014 |  | -0.0438 | -0.0852 | -0.0023 | 0.1370 | 0.0114 |
| Cd | Protein | Trunk Bone | -0.0349 | -0.0763 | 0.0065 |  | -0.0089 | -0.0144 | -0.0034 |  | -0.0438 | -0.0852 | -0.0023 | 0.2028 | 0.0016 |
| Cd | Fat | Trunk Bone | -0.0372 | -0.0785 | 0.0041 |  | -0.0065 | -0.0115 | -0.0016 |  | -0.0438 | -0.0852 | -0.0023 | 0.1494 | 0.0101 |


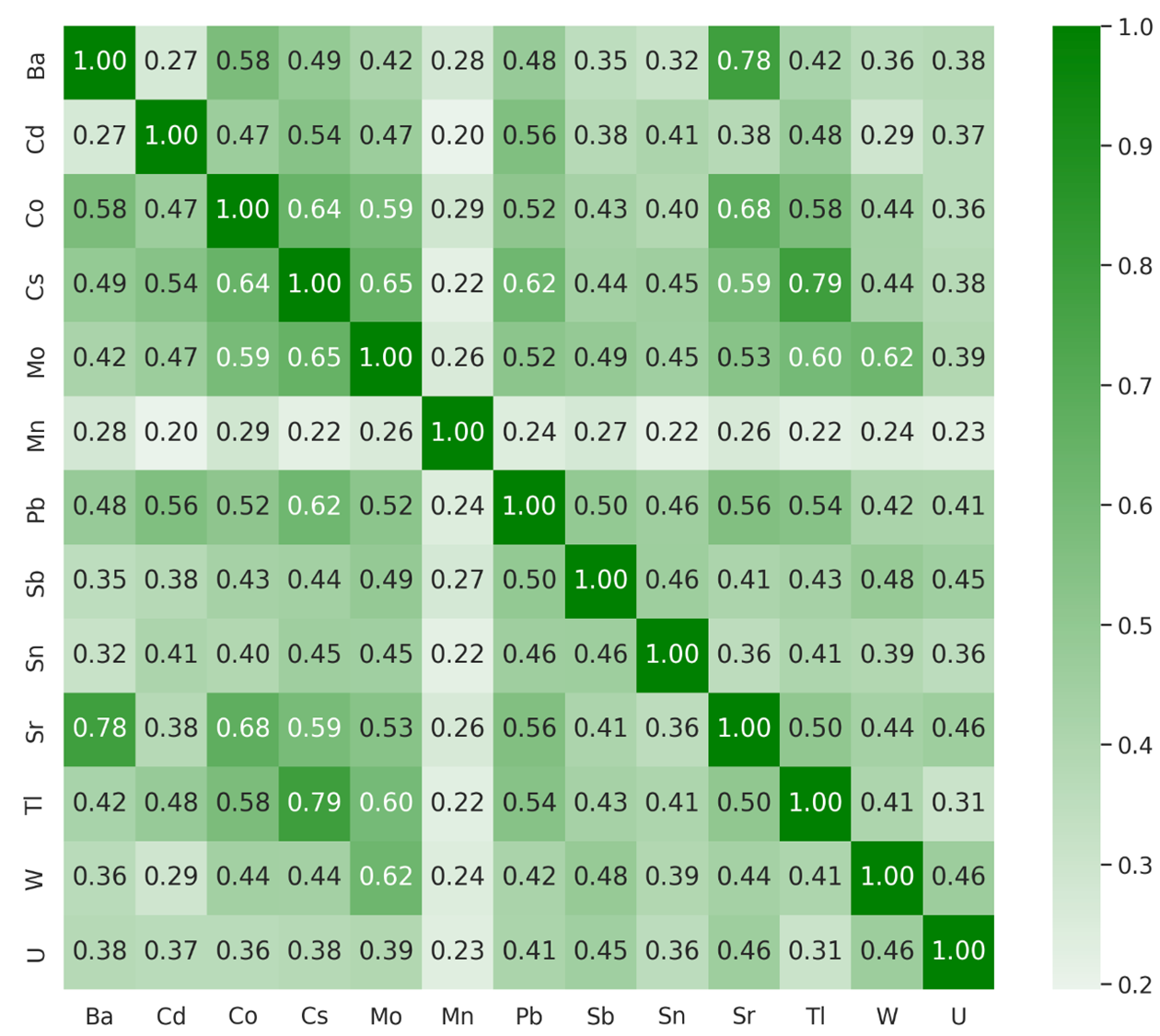


Figure S1 Pearson correlations among 9 heavy metals


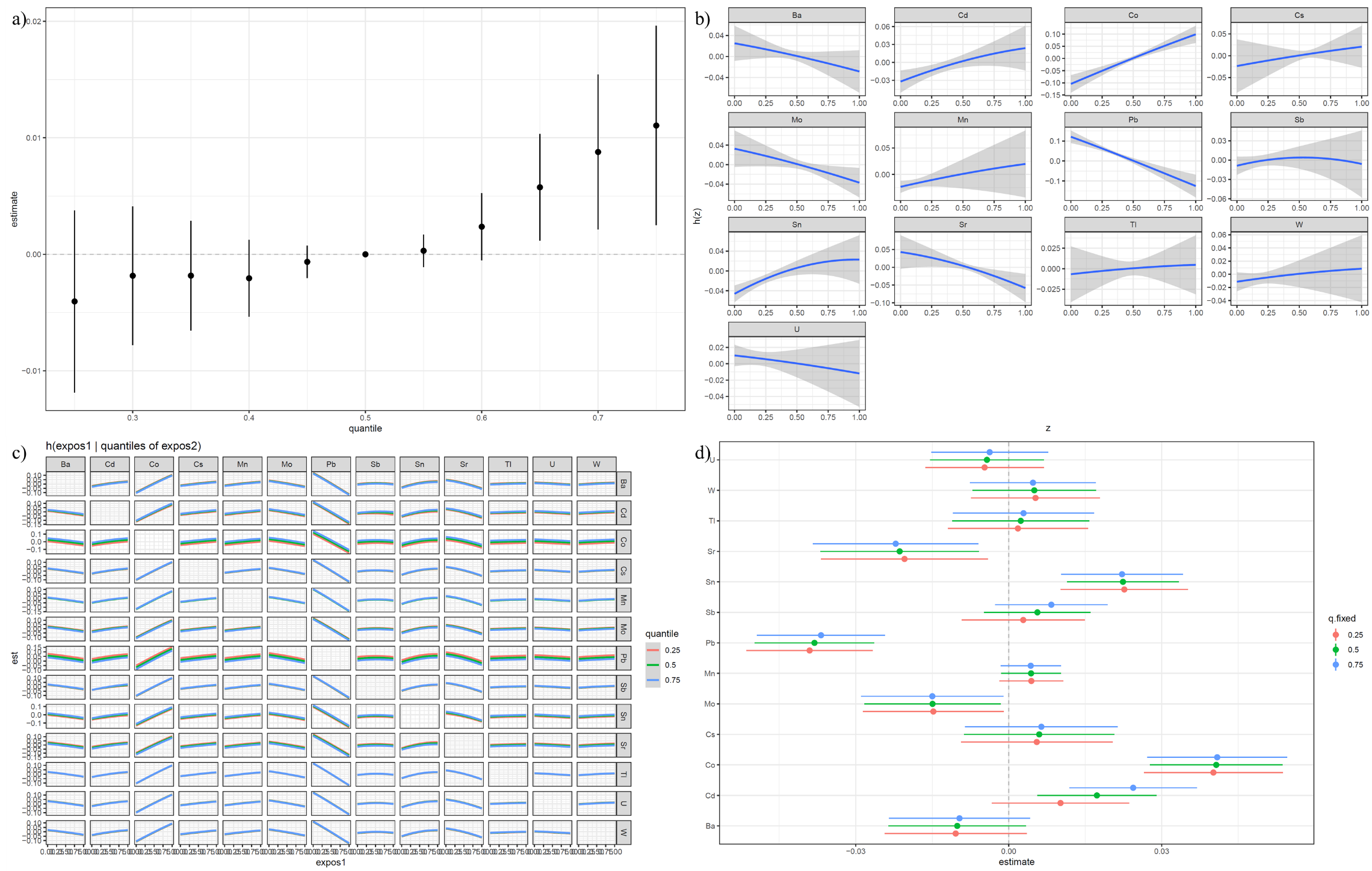


Figure S2 Association between Mixed Heavy Metal and Head Bone BMD Assessed by BKMR Model

*Note: Fig S2a is overall effect of heavy metals mixtures on BMD in BKMR model where all heavy metals at specific percentiles were compared to their 50th percentile. Fig S2b is univariate exposure–response function between each heavy metal and BMD when the other heavy metals were fixed at 50th percentiles. Fig S2c is single exposure-response functions for each heavy metal and BMD when a single heavy metal was at the 75th compared with the 50th percentile and the concentrations of all the other heavy metals were fixed at either the 25th, 50th, 75th percentile in the BKMR model. Fig S2d is bivariate exposure-response functions for each heavy metal and BMD when one heavy metal was fixed at 25th, 50th, 75th percentiles and other heavy metals were fixed at the median in the BKMR model.*


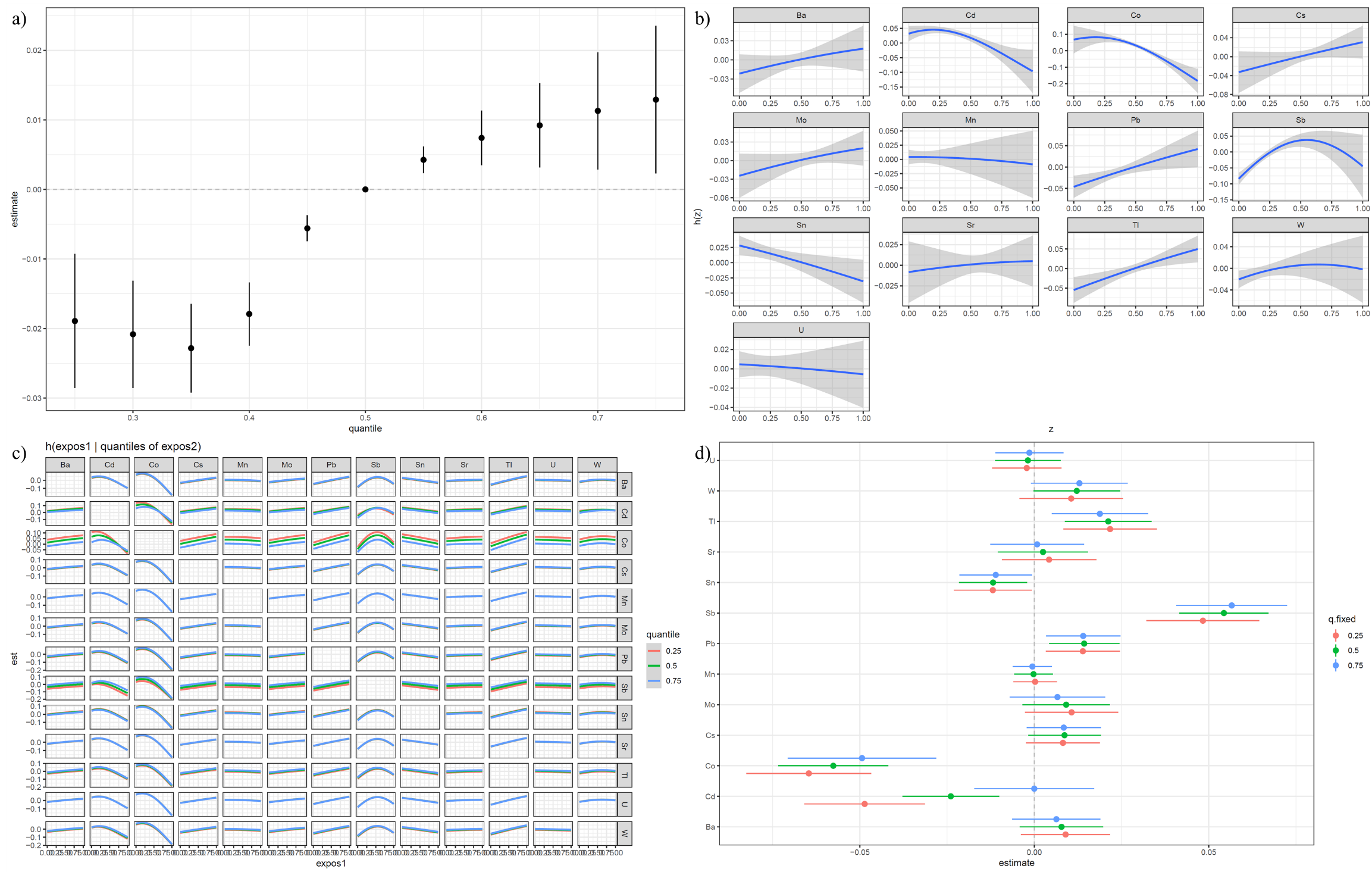


Figure S3 Association between Mixed Heavy Metal and Left Arm BMD Assessed by BKMR Model

*Note: Fig S3a is overall effect of heavy metals mixtures on BMD in BKMR model where all heavy metals at specific percentiles were compared to their 50th percentile. Fig S3b is univariate exposure–response function between each heavy metal and BMD when the other heavy metals were fixed at 50th percentiles. Fig S3c is single exposure-response functions for each heavy metal and BMD when a single heavy metal was at the 75th compared with the 50th percentile and the concentrations of all the other heavy metals were fixed at either the 25th, 50th, 75th percentile in the BKMR model. Fig S3d is bivariate exposure-response functions for each heavy metal and BMD when one heavy metal was fixed at 25th, 50th, 75th percentiles and other heavy metals were fixed at the median in the BKMR model.*


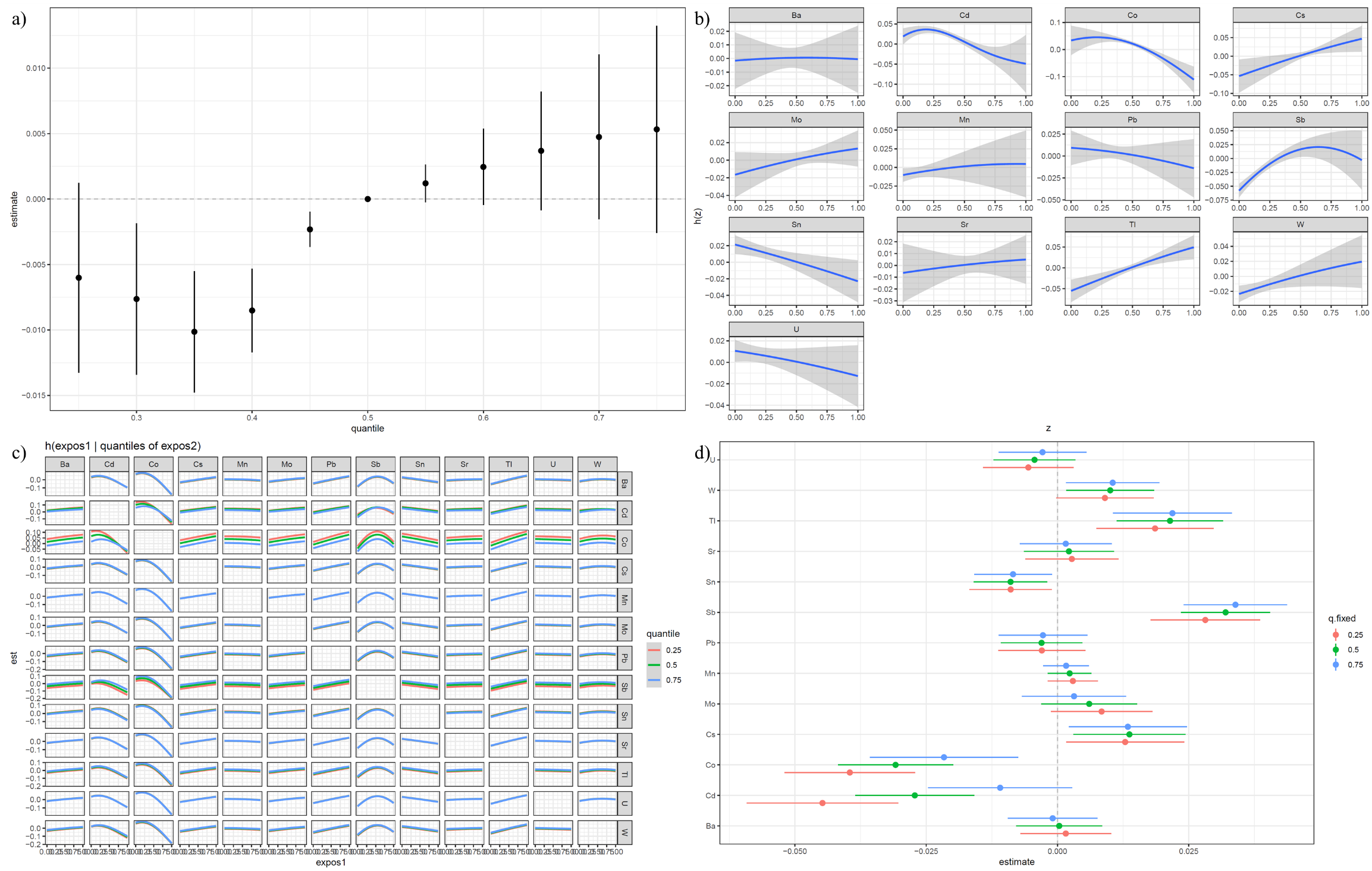


Figure S4 Association between Mixed Heavy Metal and Left Leg BMD Assessed by BKMR Model

*Note: Fig S4a is overall effect of heavy metals mixtures on BMD in BKMR model where all heavy metals at specific percentiles were compared to their 50th percentile. Fig S4b is univariate exposure–response function between each heavy metal and BMD when the other heavy metals were fixed at 50th percentiles. Fig S4c is single exposure-response functions for each heavy metal and BMD when a single heavy metal was at the 75th compared with the 50th percentile and the concentrations of all the other heavy metals were fixed at either the 25th, 50th, 75th percentile in the BKMR model. Fig S4d is bivariate exposure-response functions for each heavy metal and BMD when one heavy metal was fixed at 25th, 50th, 75th percentiles and other heavy metals were fixed at the median in the BKMR model.*


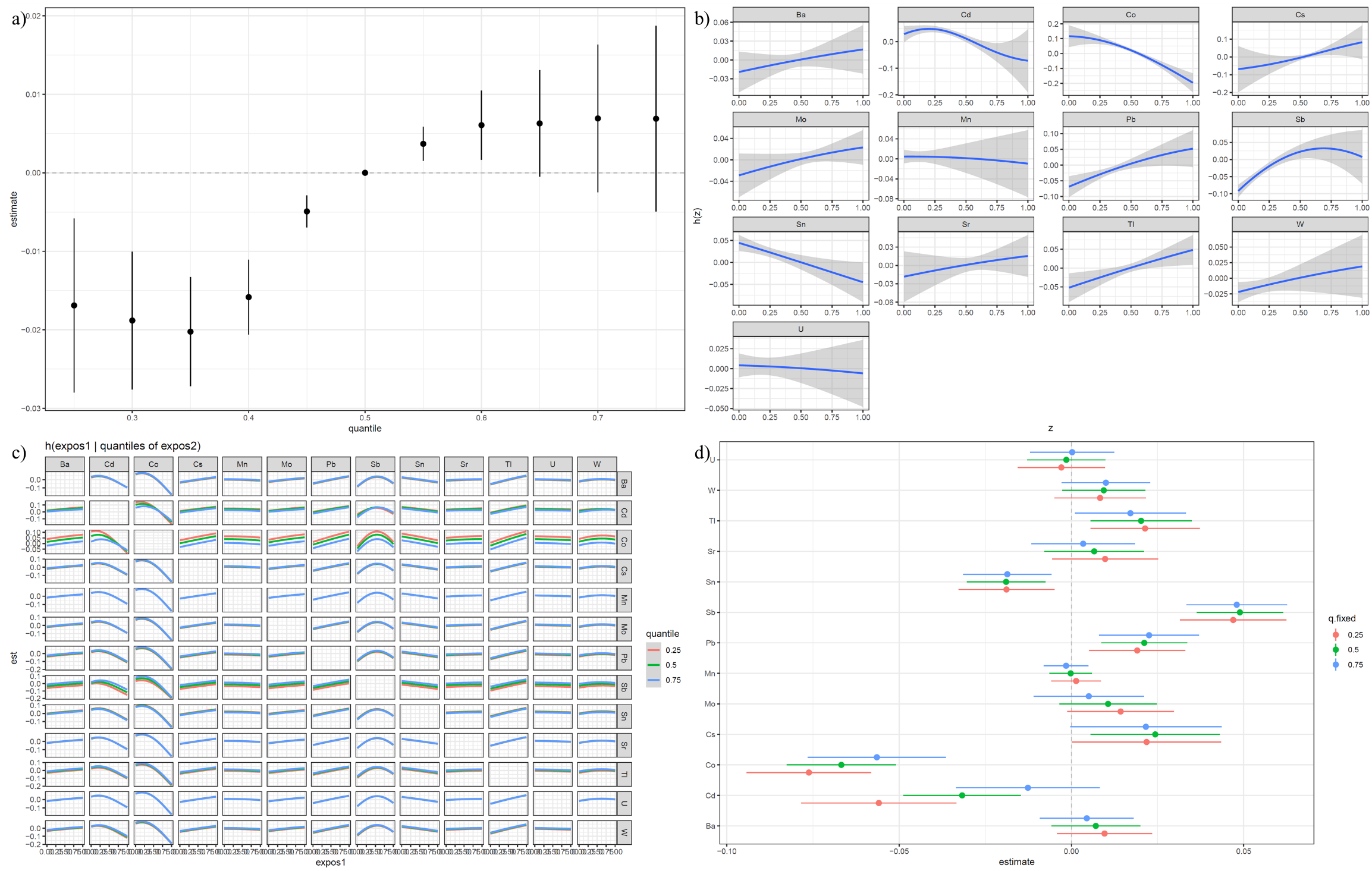


Figure S5 Association between Mixed Heavy Metal and Right Arm BMD Assessed by BKMR Model

*Note: Fig S5a is overall effect of heavy metals mixtures on BMD in BKMR model where all heavy metals at specific percentiles were compared to their 50th percentile. Fig S5b is univariate exposure–response function between each heavy metal and BMD when the other heavy metals were fixed at 50th percentiles. Fig S5c is single exposure-response functions for each heavy metal and BMD when a single heavy metal was at the 75th compared with the 50th percentile and the concentrations of all the other heavy metals were fixed at either the 25th, 50th, 75th percentile in the BKMR model. Fig S5d is bivariate exposure-response functions for each heavy metal and BMD when one heavy metal was fixed at 25th, 50th, 75th percentiles and other heavy metals were fixed at the median in the BKMR model.*


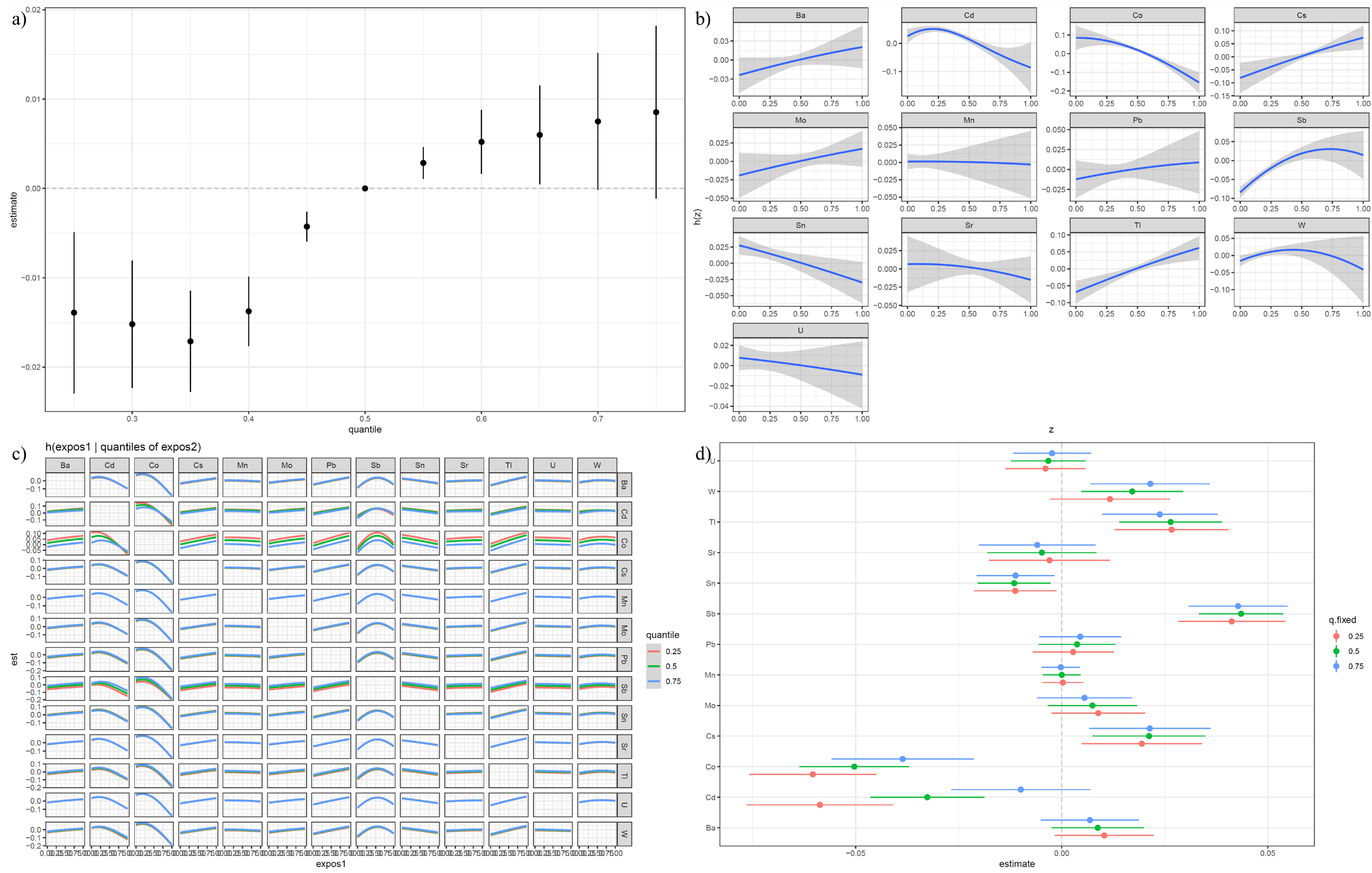


Figure S6 Association between Mixed Heavy Metal and Right Leg BMD Assessed by BKMR Model

*Note: Fig S6a is overall effect of heavy metals mixtures on BMD in BKMR model where all heavy metals at specific percentiles were compared to their 50th percentile. Fig S6b is univariate exposure–response function between each heavy metal and BMD when the other heavy metals were fixed at 50th percentiles. Fig S6c is single exposure-response functions for each heavy metal and BMD when a single heavy metal was at the 75th compared with the 50th percentile and the concentrations of all the other heavy metals were fixed at either the 25th, 50th, 75th percentile in the BKMR model. Fig S6d is bivariate exposure-response functions for each heavy metal and BMD when one heavy metal was fixed at 25th, 50th, 75th percentiles and other heavy metals were fixed at the median in the BKMR model.*


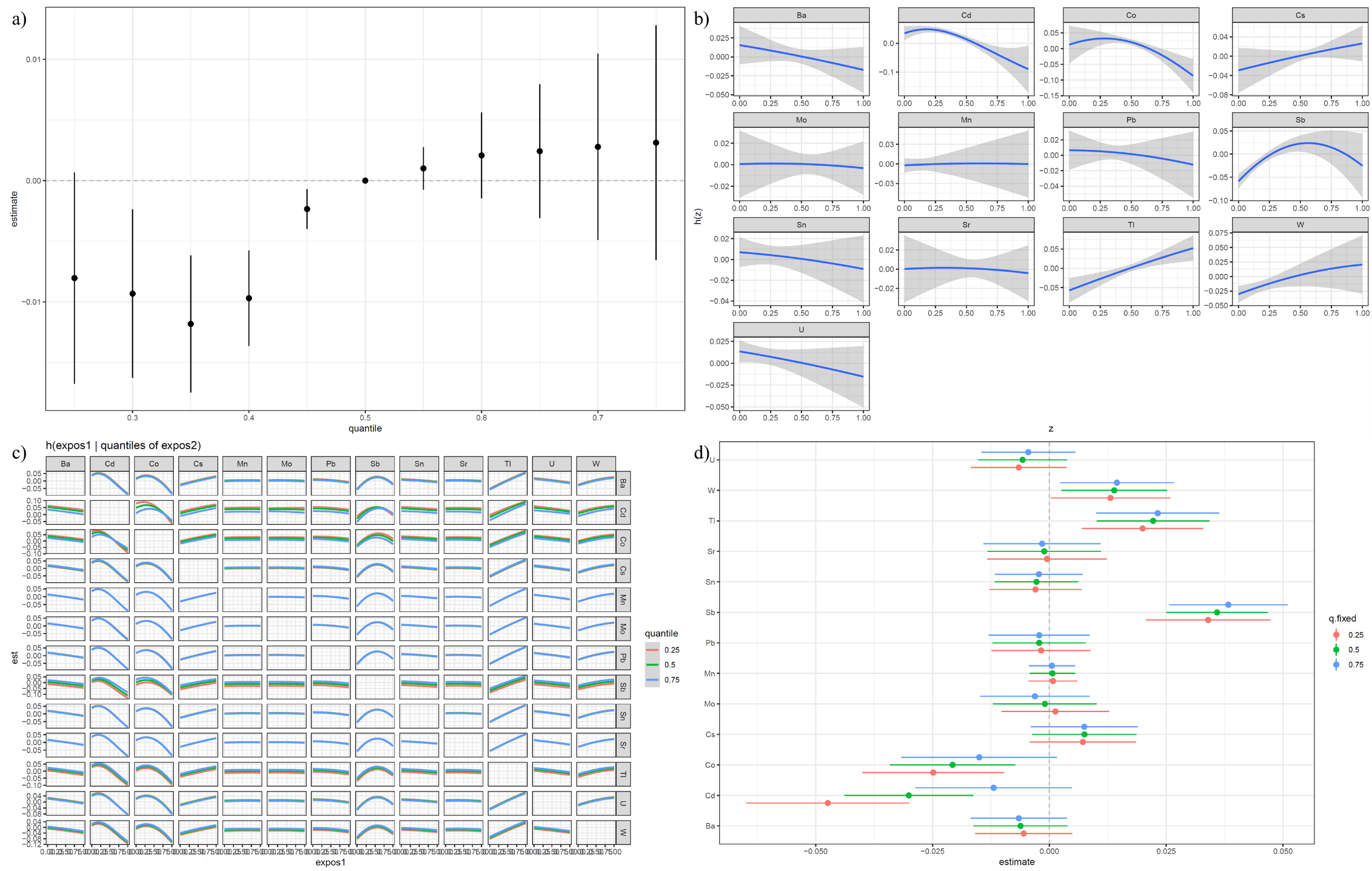


Figure S7 Association between Mixed Heavy Metal and Left Rib BMD Assessed by BKMR Model

*Note: Fig S7a is overall effect of heavy metals mixtures on BMD in BKMR model where all heavy metals at specific percentiles were compared to their 50th percentile. Fig S7b is univariate exposure–response function between each heavy metal and BMD when the other heavy metals were fixed at 50th percentiles. Fig S7c is single exposure-response functions for each heavy metal and BMD when a single heavy metal was at the 75th compared with the 50th percentile and the concentrations of all the other heavy metals were fixed at either the 25th, 50th, 75th percentile in the BKMR model. Fig S7d is bivariate exposure-response functions for each heavy metal and BMD when one heavy metal was fixed at 25th, 50th, 75th percentiles and other heavy metals were fixed at the median in the BKMR model.*


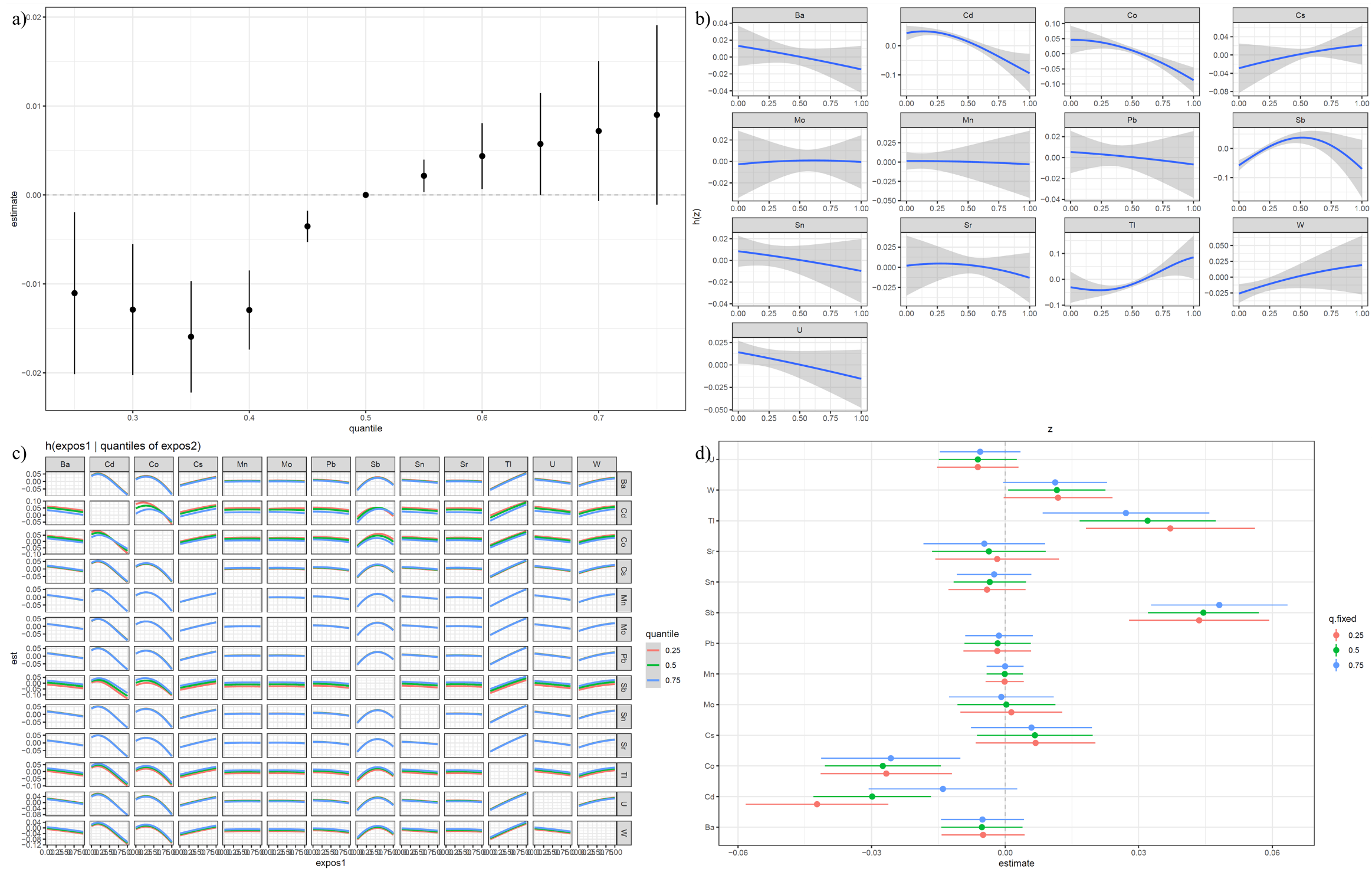


Figure S8 Association between Mixed Heavy Metal and Right Rib BMD Assessed by BKMR Model

*Note: Fig S8a is overall effect of heavy metals mixtures on BMD in BKMR model where all heavy metals at specific percentiles were compared to their 50th percentile. Fig S8b is univariate exposure–response function between each heavy metal and BMD when the other heavy metals were fixed at 50th percentiles. Fig S8c is single exposure-response functions for each heavy metal and BMD when a single heavy metal was at the 75th compared with the 50th percentile and the concentrations of all the other heavy metals were fixed at either the 25th, 50th, 75th percentile in the BKMR model. Fig S8d is bivariate exposure-response functions for each heavy metal and BMD when one heavy metal was fixed at 25th, 50th, 75th percentiles and other heavy metals were fixed at the median in the BKMR model.*


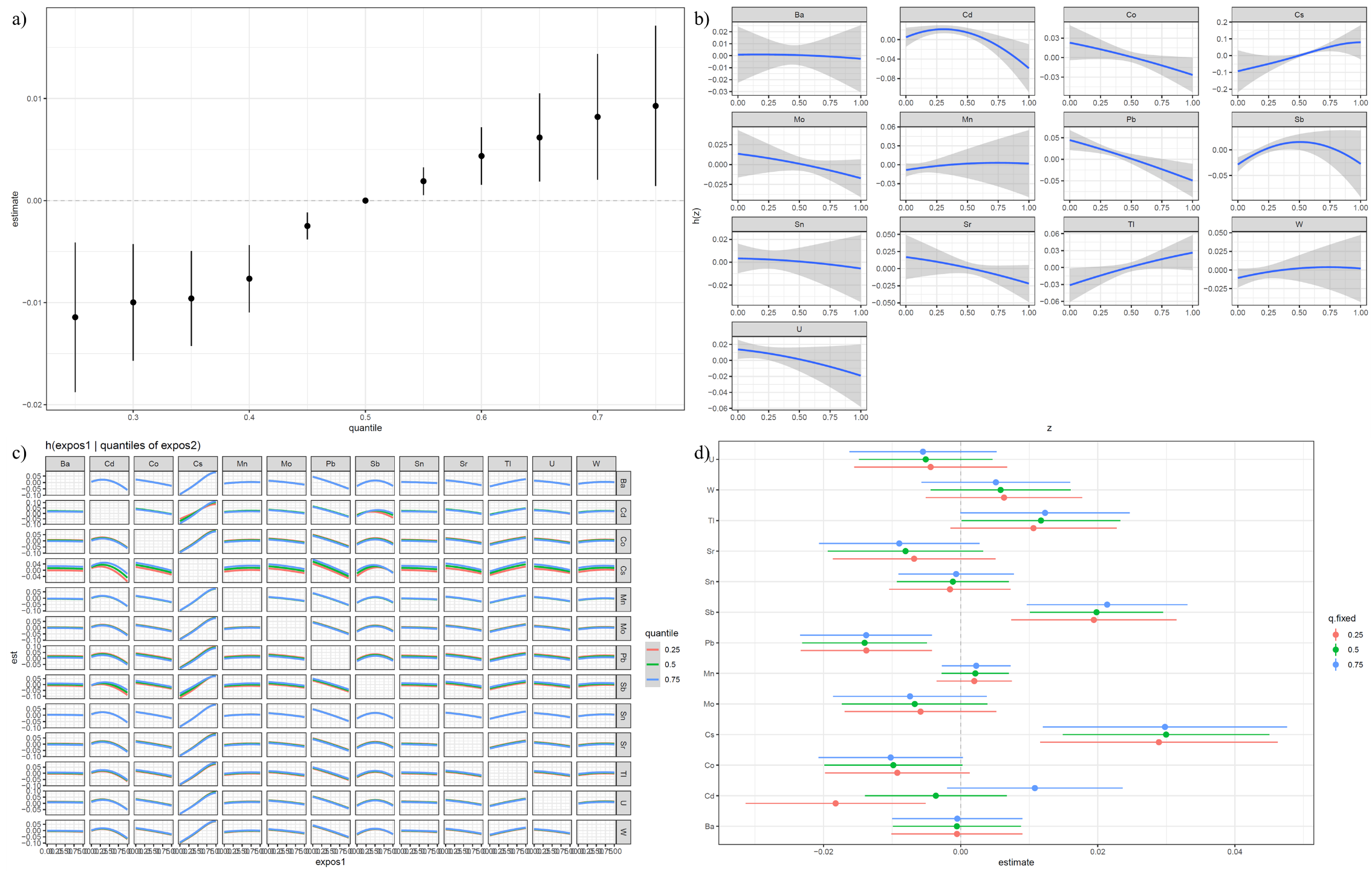


Figure S9 Association between Mixed Heavy Metal and Thoracic Spine BMD Assessed by BKMR Model

*Note: Fig S9a is overall effect of heavy metals mixtures on BMD in BKMR model where all heavy metals at specific percentiles were compared to their 50th percentile. Fig S9b is univariate exposure–response function between each heavy metal and BMD when the other heavy metals were fixed at 50th percentiles. Fig S9c is single exposure-response functions for each heavy metal and BMD when a single heavy metal was at the 75th compared with the 50th percentile and the concentrations of all the other heavy metals were fixed at either the 25th, 50th, 75th percentile in the BKMR model. Fig S9d is bivariate exposure-response functions for each heavy metal and BMD when one heavy metal was fixed at 25th, 50th, 75th percentiles and other heavy metals were fixed at the median in the BKMR model.*


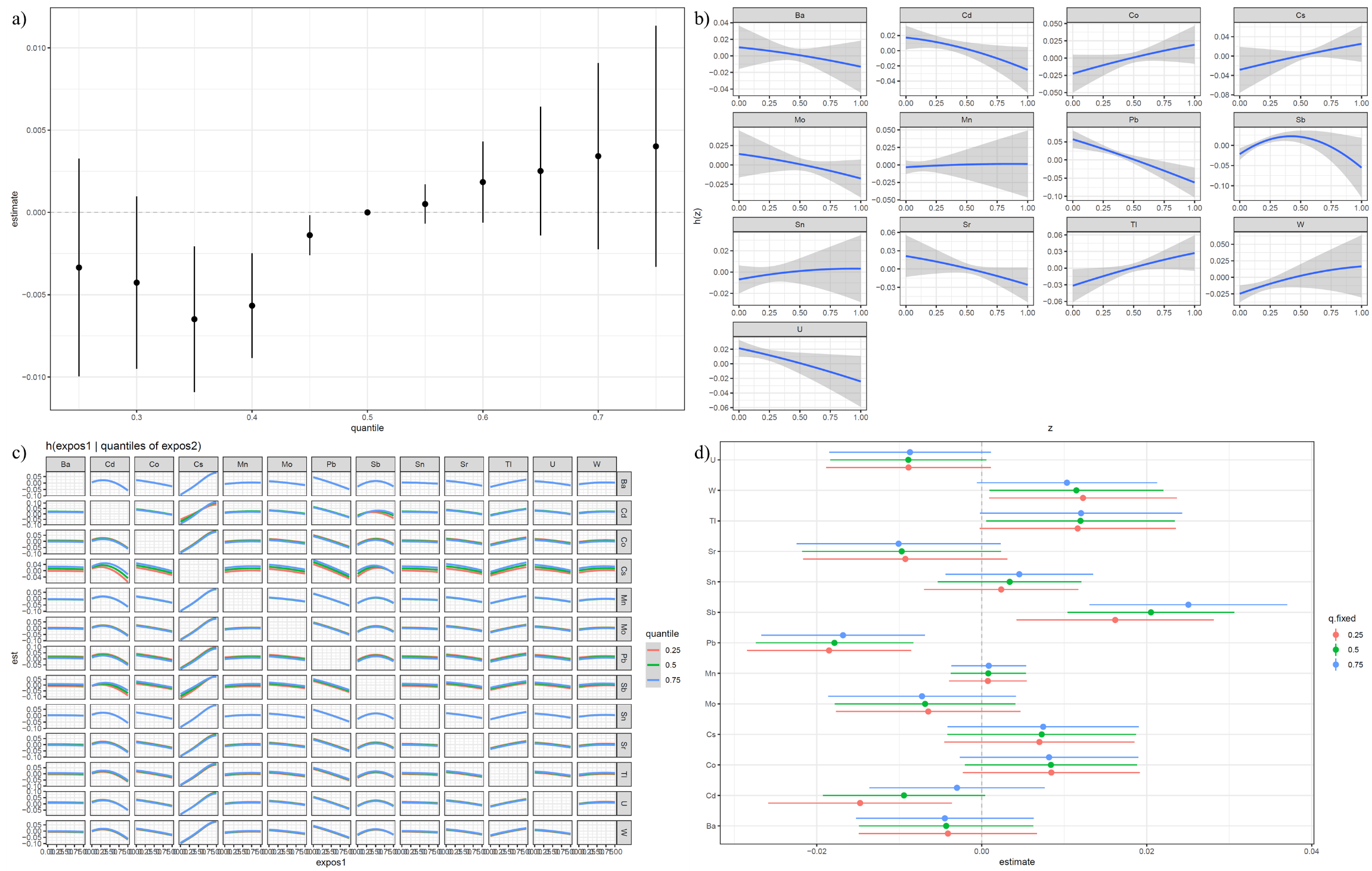


Figure S10 Association between Mixed Heavy Metal and Lumbar Spine BMD Assessed by BKMR Model

*Note: Fig S10a is overall effect of heavy metals mixtures on BMD in BKMR model where all heavy metals at specific percentiles were compared to their 50th percentile. Fig S10b is univariate exposure–response function between each heavy metal and BMD when the other heavy metals were fixed at 50th percentiles. Fig S10c is single exposure-response functions for each heavy metal and BMD when a single heavy metal was at the 75th compared with the 50th percentile and the concentrations of all the other heavy metals were fixed at either the 25th, 50th, 75th percentile in the BKMR model. Fig S10d is bivariate exposure-response functions for each heavy metal and BMD when one heavy metal was fixed at 25th, 50th, 75th percentiles and other heavy metals were fixed at the median in the BKMR model.*


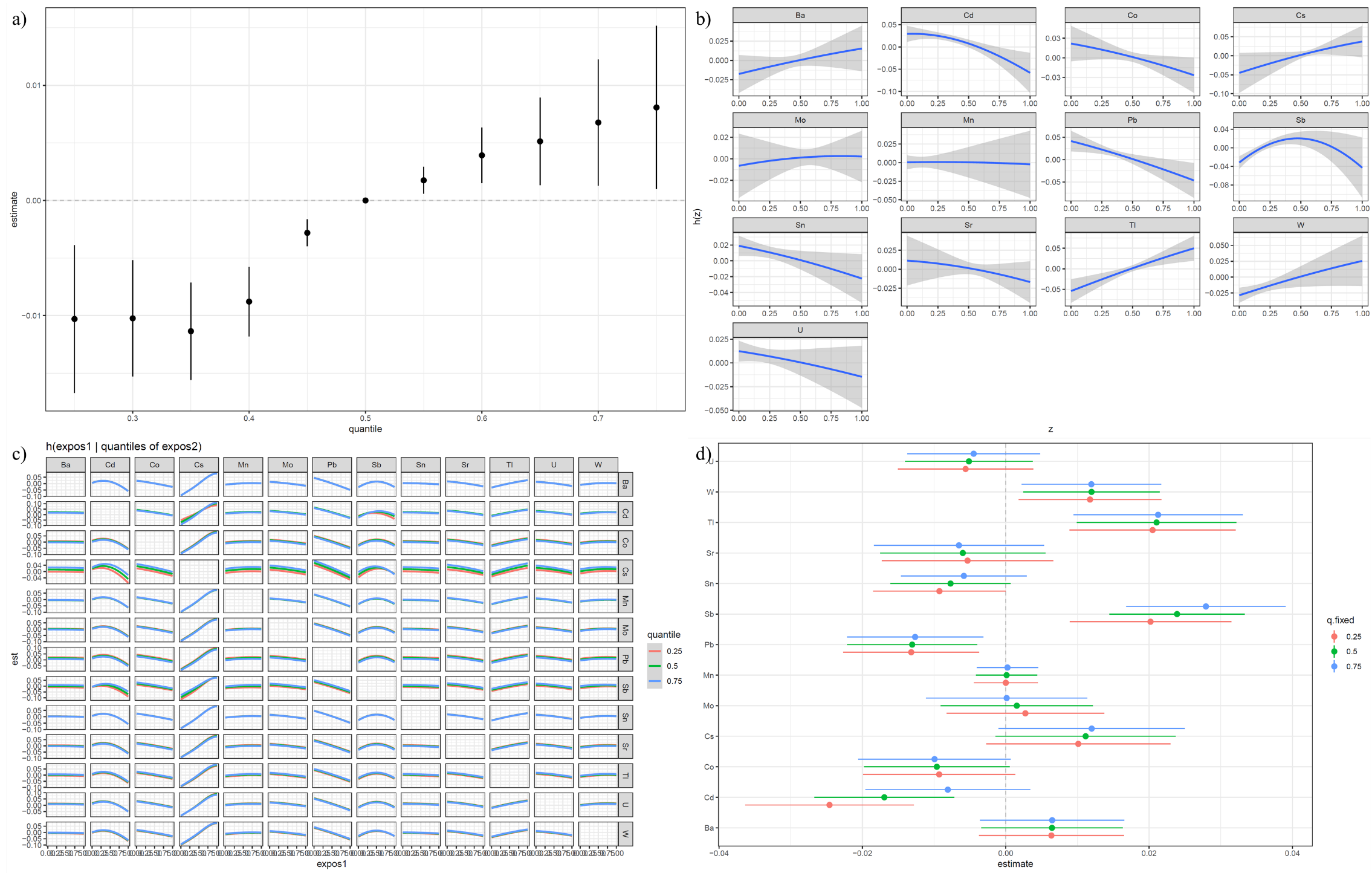


Figure S11 Association between Mixed Heavy Metal and Pelvis BMD Assessed by BKMR Model

*Note: Fig S11a is overall effect of heavy metals mixtures on BMD in BKMR model where all heavy metals at specific percentiles were compared to their 50th percentile. Fig S11b is univariate exposure–response function between each heavy metal and BMD when the other heavy metals were fixed at 50th percentiles. Fig S11c is single exposure-response functions for each heavy metal and BMD when a single heavy metal was at the 75th compared with the 50th percentile and the concentrations of all the other heavy metals were fixed at either the 25th, 50th, 75th percentile in the BKMR model. Fig S11d is bivariate exposure-response functions for each heavy metal and BMD when one heavy metal was fixed at 25th, 50th, 75th percentiles and other heavy metals were fixed at the median in the BKMR model.*


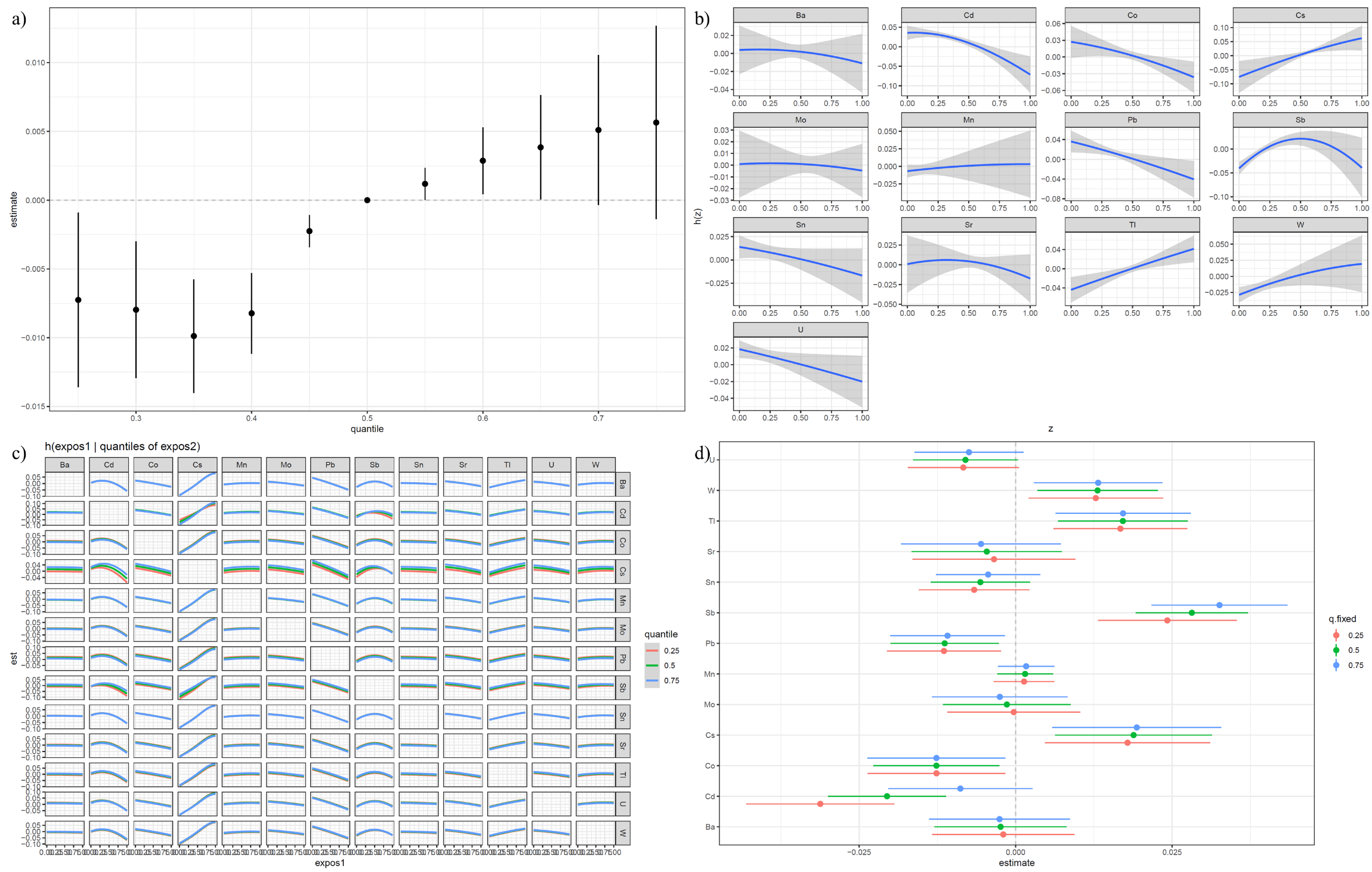


Figure S12 Association between Mixed Heavy Metal and Trunk Bone BMD Assessed by BKMR Model

*Note: Fig S12a is overall effect of heavy metals mixtures on BMD in BKMR model where all heavy metals at specific percentiles were compared to their 50th percentile. Fig S12b is univariate exposure–response function between each heavy metal and BMD when the other heavy metals were fixed at 50th percentiles. Fig S12c is single exposure-response functions for each heavy metal and BMD when a single heavy metal was at the 75th compared with the 50th percentile and the concentrations of all the other heavy metals were fixed at either the 25th, 50th, 75th percentile in the BKMR model. Fig S12d is bivariate exposure-response functions for each heavy metal and BMD when one heavy metal was fixed at 25th, 50th, 75th percentiles and other heavy metals were fixed at the median in the BKMR model.*
